# Coaggregation of RNA-Binding Proteins in a Model of TDP-43 Proteinopathy with Selective RGG Motif Methylation and a Role for RRM1 Ubiquitination

**DOI:** 10.1371/journal.pone.0038658

**Published:** 2012-06-21

**Authors:** Eric B. Dammer, Claudia Fallini, Yair M. Gozal, Duc M. Duong, Wilfried Rossoll, Ping Xu, James J. Lah, Allan I. Levey, Junmin Peng, Gary J. Bassell, Nicholas T. Seyfried

**Affiliations:** 1 Department of Human Genetics, Emory University School of Medicine, Atlanta, Georgia, United States of America; 2 Department of Cell Biology, Emory University School of Medicine, Atlanta, Georgia, United States of America; 3 Department of Neurology, Emory University School of Medicine, Atlanta, Georgia, United States of America; 4 Department of Biochemistry, Emory University School of Medicine, Atlanta, Georgia, United States of America; 5 Center for Neurodegenerative Diseases, Emory University School of Medicine, Atlanta, Georgia, United States of America; Thomas Jefferson University, United States of America

## Abstract

TAR DNA-binding protein 43 (TDP-43) is a major component within ubiquitin-positive inclusions of a number of neurodegenerative diseases that increasingly are considered as TDP-43 proteinopathies. Identities of other inclusion proteins associated with TDP-43 aggregation remain poorly defined. In this study, we identify and quantitate 35 co-aggregating proteins in the detergent-resistant fraction of HEK-293 cells in which TDP-43 or a particularly aggregate prone variant, TDP-S6, were enriched following overexpression, using stable isotope-labeled (SILAC) internal standards and liquid chromatography coupled to tandem mass spectrometry (LC-MS/MS). We also searched for differential post-translational modification (PTM) sites of ubiquitination. Four sites of ubiquitin conjugation to TDP-43 or TDP-S6 were confirmed by dialkylated GST-TDP-43 external reference peptides, occurring on or near RNA binding motif (RRM) 1. RRM-containing proteins co-enriched in cytoplasmic granular structures in HEK-293 cells and primary motor neurons with insoluble TDP-S6, including cytoplasmic stress granule associated proteins G3BP, PABPC1, and eIF4A1. Proteomic evidence for TDP-43 co-aggregation with paraspeckle markers RBM14, PSF and NonO was also validated by western blot and by immunocytochemistry in HEK-293 cells. An increase in peptides from methylated arginine-glycine-glycine (RGG) RNA-binding motifs of FUS/TLS and hnRNPs was found in the detergent-insoluble fraction of TDP-overexpressing cells. Finally, TDP-43 and TDP-S6 detergent-insoluble species were reduced by mutagenesis of the identified ubiquitination sites, even following oxidative or proteolytic stress. Together, these findings define some of the aggregation partners of TDP-43, and suggest that TDP-43 ubiquitination influences TDP-43 oligomerization.

## Introduction

TDP-43 is a major protein component in ubiquitin-positive, tau- and α-synuclein-negative inclusions of frontotemporal lobar degeneration (FTLD) and amyotrophic lateral sclerosis (ALS) [1], [2], which was initially identified due to its specific enrichment in the detergent-insoluble biochemical fraction of FTLD frontal cortex [1]. Although physiological TDP-43 is a predominantly nuclear protein with the capacity to transiently shuttle to and from the cytoplasm in a manner dependent upon general transcription [3], pathological TDP-43 redistributes from the nucleus to the cytoplasm where it more often aggregates following phosphorylation, ubiquitination and proteolytic cleavage [1], [2], [4], [5]. Despite recent progress in demonstrating that TDP-43 C-terminal fragments aggregate in cytoplasm in relative absence of RNA or dynein-dependent transport [6], a comprehensive understanding of molecular mechanisms that determine or ensue from TDP-43 aggregation remains elusive. Cultured neurons and HEK-293 cells expressing full length TDP-43 consistently localize the protein almost exclusively to the nucleus [7]. In contrast, a potential naturally occurring human 33.5 kDa N-terminal splicing variant of TDP-43 (TDP-S6) displays prominent cytoplasmic aggregation and post-translational modification (PTM) upon over-expression [7], recapitulating disease phenotype. The splicing event leading to TDP-S6 mRNA detected in mouse (encoding the protein with Uniprot ID C9DT14) skips the large, evolutionarily conserved 5′ exon encoding the glycine-rich C-terminus of full length TDP-43 which is itself responsible for promoting exon skipping events in splicing [8], and leads to utilization of a highly conserved alternative exon with a premature stop codon. Like the mouse isoform, human TDP-S6 has 18 unique amino acids at its C-terminus and is 295 residues in total compared to the full length protein with 414 residues. The detergent-insoluble biochemical fraction for TDP-S6 expressing cells accumulated ubiquitin and SUMO2 or SUMO3 (SUMO2/3) conjugates at high-molecular weights, whereas the same fraction from TDP-43 overexpressing cells showed only an increase in SUMO2/3. Compared to TDP-S6, TDP-43 was more robustly phosphorylated in western blots, consistent with phosphorylation on two C-terminal serine residues only present in TDP-43 [7], [9].

It is unknown if TDP-43- or TDP-S6-associated post-translational modifications (PTMs) play a role in the mechanism(s) underlying TDP-43 proteinopathy because precise identification of PTM sites and PTM involvement in recruiting interaction partners to biochemically insoluble aggregates with TDP-43 remains largely unexplored. Mechanisms of TDP-43 aggregation defined in cellular models, particularly determinants in terms of primary structure motifs and PTMs on TDP-43 or partners could provide insight into pathology in more complex tissues. PTMs generally influence protein-protein, protein-nucleic acid, and/or protein-membrane interactions by altering or augmenting the protein surfaces available for stable interactions with select PTM-sensitive or PTM-dependent partners. For example, ubiquitination of a protein can enable interactions with ubiquitin receptors facilitating aggresome formation, or with other receptors that drive protein flux through the proteasome, or at autophagosomes accumulating proteins for degradation [10].

**Figure 1:**
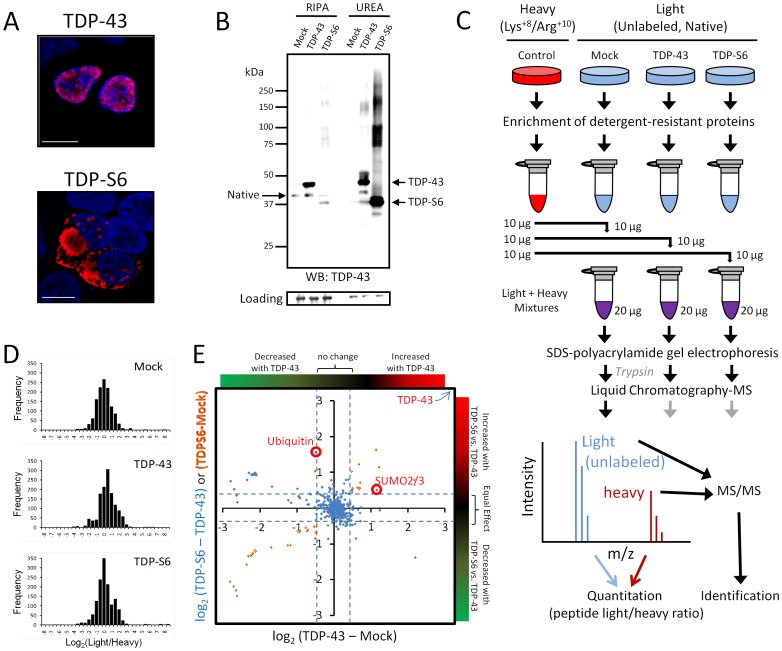
Characterization of the aggregate proteome of HEK-293 cells overexpressing TDP-43 and aggregate prone TDP-S6. (**A**) HEK-293 cells were transfected with HA-TDP-43 or HA-TDP-S6 and immunostained with anti-HA antibody for recombinant TDP-43 exclusively (red) and Hoescht stain for the nucleus (blue). Scale bars, 10 µm. (**B**) Western blotting (WB) of native TDP-43 in the detergent soluble (RIPA) and insoluble (urea) fractions from mock, TDP-43 and TDP-S6 transfected HEK-293 cells. 10 µg and 5 µg of detergent-soluble and insoluble sample were loaded, respectively. A ponceau S nonspecific band was used to show equal loading. (**C**) Workflow for quantitative proteomics using isotopic labeled internal standards. In this approach, detergent insoluble urea extracts from isotopically-labeled control HEK-293 cells are mixed equally with urea extracts prepared from mock, TDP-43 and TDP-S6 transfected cells. The samples are resolved on an SDS-PAGE gel, excised into gel slices, digested with trypsin, and analyzed by liquid chromatography coupled with tandem mass spectrometry (LC-MS/MS) on a high-resolution Orbitrap mass spectrometer. The isotopically labeled peptides from the control HEK-293 cells are chemically identical to their unlabeled counterparts and serve as internal standards for the measurement of protein abundance across samples. (**D**) Histograms of the entire population of quantified proteins expressed as log_2_(light/heavy) vs. frequency for the three mixtures with Mock (null experiment), TDP-43, and TDP-S6. (**E**) To visualize proteins significantly changing in the TDP-43 and/or TDP-S6 models, we constructed a triple SILAC map of the differences in log_2_-transformed ratios. Confirming high transfection efficiency, we identified both TDP-43 and TDP-S6 as the most enriched protein component in the cell model (at the top right).

The purpose of this study was to determine what interactions with TDP-43 or TDP-S6 occur in detergent-resistant protein aggregates to suggest aggregation-associated TDP-43 function, and to unambiguously identify associated PTMs including TDP-43 ubiquitination sites. Biochemical fractionation preceding quantitative mass spectrometry was followed by extensive validation of TDP-43 or TDP-S6 overexpression-induced cytoplasmic detergent-insoluble co-aggregate proteins. We also characterized similarly composed arsenite-induced TDP-43-positive cytoplasmic stress granules and spontaneous TDP-S6 inclusions in both HEK-293 cells and primary motor neurons using immunofluorescence colocalization. Four ubiquitination sites on TDP-43 or TDP-S6 were identified in addition to TDP-43 overexpression-induced methylation on intrinsically detergent insoluble proteins. An intriguing and unexpected finding is that the methylation and ubiquitination events that co-occur during TDP-43 or TDP-S6 overexpression occur primarily on RNA interaction motifs, suggesting that these PTMs play a role in remodeling the network of cellular RNPs during protein aggregation which may be an underlying process in neurodegenerative proteinopathy.

**Table 1:** Proteins significantly changed in the TDP-43 and TDP-S6 aggregate proteome.

				Quantified Peptides^1^			Log_2_ Ratio Difference^2^			Prev. ReportedInteraction
NCBI Reference	Description	Gene Symbol		Mock	TDP43	TDPS6	43-Mock	S6-Mock	S6–43	
	***TDP-43 and TDP-S6***									
NP_031401.1	TAR DNA-binding protein 43	TARDBP* ^,Λ^	↑	9	26	26	3.51	6.32	2.81	√
NP_001005849.1	small ubiquitin-related modifier 2 isoform b precursor	SUMO2	↑	2	4	4	1.64	1.95	0.31	
NP_006319.1	RNA-binding protein 14	RBM14*	↑	11	14	14	1.18	1.55	0.37	X
NP_006784.1	thioredoxin-dependent peroxide reductase, mitochondrial isoform a precursor	PRDX3	↑	7	7	2	0.98	1.34	0.36	
NP_006749.1	splicing factor U2AF 35 kDa subunit isoform a	U2AF1* ^,Λ^	↑	2	2	2	0.96	1.26	0.30	
NP_006796.1	heterogeneous nuclear ribonucleoprotein A0	HNRNPA0* ^, Λ^	↑	10	10	9	1.21	1.03	−0.18	√
NP_004387.1	probable ATP-dependent RNA helicase DDX5	DDX5^Λ^	↑	28	34	23	1.14	0.89	−0.25	√
NP_004915.2	alpha-actinin-4	ACTN4	↓	66	36	36	−2.32	−1.76	0.56	
NP_005955.1	myosin-10	MYH10	↓	136	71	109	−1.87	−1.28	0.59	
NP_001605.1	actin, cytoplasmic 2	ACTG1	↓	56	54	49	−1.83	−1.04	0.79	
NP_001035203.1	myosin-11 isoform SM1B	MYH11	↓	25	11	23	−1.63	−0.90	0.73	
	***TDP-S6***									
NP_005745.1	Ras GTPase-activating protein-binding protein 1	G3BP1* ^, Λ^	↑	7	7	12	0.28†	1.70	1.43	√
NP_002945.1	ubiquitin and ribosomal protein S27a precursor	RPS27A	↑	6	6	5	−0.02	1.35	1.37	√
NP_002559.2	polyadenylate-binding protein 1	PABPC1*	↑	19	19	16	0.76	1.25	0.49	√
NP_001407.1	eukaryotic initiation factor 4A-I	EIF4A1^Λ^	↑	26	33	25	0.81	1.03	0.22	√
NP_031389.3	non-POU domain-containing octamer-binding protein isoform 1; NonO	NONO*	↑	19	23	21	0.72	1.02	0.30	√
NP_006550.1	KH domain-containing, RNA-binding, signaltransduction-associated protein 1	KHDRBS1^Λ^	↑	8	4	5	0.41	0.99	0.58	X
NP_006089.1	guanine nucleotide-binding protein subunit beta-2-like 1	GNB2L1	↑	11	8	10	0.72	0.96	0.24	√
NP_001027454.1	thymopoietin isoform beta	TMPO	↑	5	6	8	0.46	0.93	0.47	
NP_004153.2	Ras-related protein Rab-5A	RAB5A	↓	2	3	4	−0.77	−1.32	−0.55	
	***TDP-43***									
NP_000960.2	60 S ribosomal protein L5	RPL5	↑	2	6	4	1.20	0.64	−0.56	√
NP_004517.2	DNA replication licensing factor MCM2	MCM2	↑, 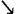	6	4	11	1.08	0.03	−1.05	
NP_001009570.1	T-complex protein 1 subunit eta isoform b	CCT7	↑	3	4	4	1.06	0.42	−0.64	
NP_005057.1	splicing factor, proline- and glutamine-rich; PTB-associated splicing factor PSF	SFPQ* ^,Λ^	↑	18	20	13	0.99	0.75	−0.24	√
NP_005234.1	RNA-binding protein EWS isoform 2	EWSR1* ^,Λ^	↑, 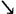	4	2	2	0.99	0.33	−0.66	
NP_000997.1	40 S ribosomal protein S3a	RPS3A	↑	7	10	4	0.98	0.59	−0.39	√
NP_002873.1	Ran-specific GTPase-activating protein	RANBP1	↑	5	5	6	0.97	0.76	−0.21	
NP_003964.3	60 S ribosomal protein L14	RPL14	↑	5	6	5	0.96	0.58	−0.38	√
NP_444505.1	60 S acidic ribosomal protein P0	RPLP0	↑	8	11	8	0.95	0.54	−0.42	√
NP_006266.2	splicing factor, arginine/serine-rich 6	SRSF6*	↑	3	4	7	0.95	0.41	−0.54	
NP_036611.2	14-3-3 protein gamma	YWHAG	↑	12	18	12	0.95	0.52	−0.43	
NP_001367.2	cytoplasmic dynein 1 heavy chain 1	DYNC1H1	↑, 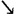	38	25	39	0.94	0.28	−0.66	
NP_000958.1	60 S ribosomal protein L3 isoform a	RPL3	↑	9	20	13	0.92	0.57	−0.35	√
NP_001460.1	X-ray repair cross-complementing protein 6	XRCC6	↑	29	39	23	0.91	0.38	−0.53	√
NP_859047.1	peroxiredoxin-1	PRDX1	↑	14	13	17	0.91	0.88	−0.04	
NP_006126.1	F-actin-capping protein subunit alpha-1	CAPZA1	↓	6	4	5	−1.20	−0.80	0.41	
	***Lost in TDP-S6*** ** ( 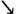 )**									
NP_057018.1	nucleolar protein 58	NOP58	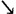	22	29	20	0.33	−0.73	−1.06	√
NP_001354.1	h/ACA ribonucleoprotein complex subunit 4 isoform 1; dyskerin 1	DKC1	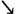	8	15	8	0.32	−0.36	−0.68	
NP_002583.1	proliferating cell nuclear antigen	PCNA	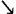	5	7	5	0.80	0.10	−0.70	
NP_006017.1	histone H1x	H1FX	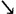	4	3	3	0.55	−0.17	−0.72	√
NP_001348.2	ATP-dependent RNA helicase A	DHX9^Λ^	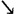	52	45	55	0.74	0.08	−0.66	√
NP_001203.1	complement component 1 Q subcomponent-binding protein precursor	C1QBP	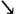	8	10	8	0.84	0.17	−0.67	√

1 Total number of peptide quantified in two independent experiments. All proteins and quantified peptides for both experiments are provided in Table S1.

2 Average from two independent experiments.

* Contains one or more RRM domains. ^∧^ Contains RGG or RGG-like motif(s).

† Quantified G3BP1 ratio difference in individual replicates is −0.15 and +0.70.

√ Previously reported soluble interaction in Freibaum, *et al* (2009) [23]. X, Additional interaction reported in Ling, et al (2010) [24].

## Materials and Methods

### Ethics Statement

C57BL6 timed pregnant mice (Charles Rivers) were sacrificed following ethical standards in the protocol approved by the Institutional Animal Care and Use Committee (IACUC) at Emory University.

### Reagents

Sodium arsenite, IU-1, and MG-132 for treatment of cultured cells were respectively obtained from Sigma (St. Louis MO), R&D Systems/Tocris (Minneapolis MN), and EMD Chemicals/Calbiochem (Gibbstown NJ). All other reagents were obtained from Sigma unless otherwise noted.

### Cell transfection and Biochemical Fractionation

Human Embryonic Kidney-293 cells (HEK-293 cells available from the American Type Culture Collection as CRL-1573) (∼1×10^8^) were mock transfected or transfected with HA-TDP-43 or HA-TDP-S6 constructs for 48 hours essentially as described previously [7]. Cells were washed twice and collected in ice cold phosphate buffered saline (PBS) and then lysed in ice cold RIPA buffer (50 mM Tris·HCl pH 7.4, 1 mM EDTA, 150 mM NaCl, 1% (v/v) Triton X-100, 1% (w/v) sodium deoxycholate, 0.1% w/v SDS). Resulting lysates were spun at 10,000×g for 30 min at 4°C to generate the detergent-soluble supernatant and insoluble pellet. Detergent insoluble pellets were dissolved in urea buffer (30 mM Tris·HCl, 8 M Urea, 2% SDS, pH 8.5). Complete protease inhibitor cocktail (Roche, Indianapolis, IN) was added to all buffers immediately prior to use. Buffers for fractionation of lysates for experiments with TDP-43 ubiquitination site mutants additionally contained 10 mM iodoacetamide (IAA) and PhosSTOP phosphatase inhibitor cocktail (Roche). Transfections were performed using Fugene6 transfection reagent (Roche) according to manufacturer’s protocol. Protein concentration for all fractions was determined by Bicinchoninic Acid (BCA) protein assay (Pierce, Rockford IL) according to the manufacturer’s instructions. HeLa cells (available from the American Type Culture Collection as CCL-2), used in place of HEK-293 cells to generate transfected lysates for supplemental data, were a kind gift of Dr. Keith Wilkinson.

### Primary motor neuron culture and transfection

Primary motor neurons from E13.5 mouse embryos were isolated, cultured, and transfected by magnetofection as previously described [11]. Monomeric red fluorescent protein mCherry was fused N-terminal to human full length TDP-43 or to TDP-S6. To induce stress granule formation, cells were treated with 0.5 mM sodium arsenite for 1 hour.

**Figure 2:**
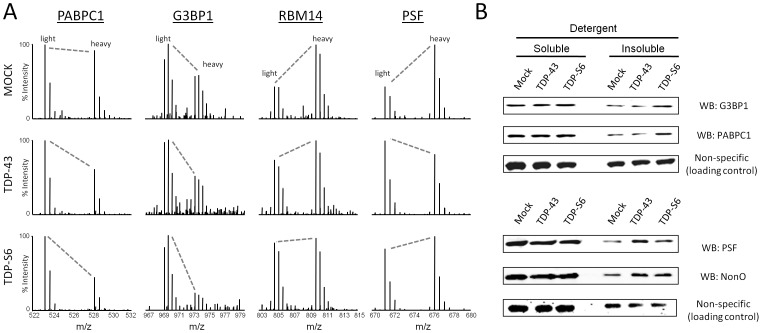
Validation of multiple RNA-binding proteins enriched within the insoluble proteome of TDP-43 and TDP-S6 transfected HEK-293 cells. (**A**) Representative quantified peptides from PABPC1, G3BP, RBM14, and PSF in the mock-transfected, TDP-43- and TDP-S6-ovexpressing detergent insoluble HEK-293 proteome with paired internal standard. (**B**) Western blot (WB) analysis for, PABPC1, G3BP, PSF and NonO in the detergent soluble (10 µg) and detergent insoluble (5 µg) proteome of transfected HEK-293 cells. A non-specific band is shown as a control to confirm equal loading.

### Immunocytochemistry and Antibodies

HEK-293 cells were cultured in Dulbecco’s Modified Eagle’s medium (Cambrex, Walkersville MD), and supplemented with 10% fetal bovine serum (Gibco, Grand Island NY) and 1% penicillin-streptomycin (Cambrex). Cells were grown in a humidified 5% CO_2_ environment at 37°C, and prepared for transfection by plating on Matrigel-coated coverslips. For overexpressing cells, immunocytochemistry was performed 48 to 60 hours after transfection as previously described [12], and cells were often passed 12–24 h before imaging. Primary antibodies included: mouse anti-TDP-43 clone 3H8 (1∶1000, Abcam, Princeton NJ) (as indicated for stress granule localization of endogenous or overexpressed TDP-43; see notes in below paragraph), anti-HA.11 mAb (1∶1000, Covance, Princeton NJ) for tagged, overexpressed TDP-43 and TDP-S6 visualization, rabbit anti-G3BP (1∶100, Proteintech Group), rabbit anti-poly(A) binding protein 1, cytoplasmic antibody (1∶30, Abcam), rabbit anti-PSF antibody (1∶100, Abcam), rabbit anti-RBM14 (1∶100, Sigma), rabbit anti-eIF4A1 (1∶100, Abcam), rabbit anti-asymmetric dimethyl arginine (1∶25, Abcam), rabbit anti-hnRNP A0 (1∶50, Abcam), rabbit anti-phospho-S409/410-2 TDP-43 (Cosmo Bio Co. Ltd., Tokyo Japan), and rabbit anti-calnexin (Enzo Life Sciences, Farmingdale NY) as a loading control. DyLight 549 and Alexa 488 fluorophore-conjugated cross-adsorbed secondary antibodies were purchased from Pierce and Jackson Immunoresearch (West Grove PA), respectively. Images were captured with a 1–2 µm optical thickness on a LSM 510 confocal microscope (Zeiss, Thornwood NY) or an Olympus BX51 fluorescent microscope for subsequent analysis. Multichannel composite images were assembled and adjusted using Photoshop CS4 (Adobe, San Jose CA).

In one experiment, −20°C methanol fixation for 2 min benefited the visualization of G3BP and TDP-43 positive stress granules with a N-terminal specific monoclonal TDP-43 antibody (1∶200, 60019-2-LG from Proteintech Group), but may have also decreased the signal from nuclear TDP species. For consistency with previous staining using paraformaldehyde fixation, all other visualization of stress granules by immunocytochemistry was performed with paraformaldehyde, although like the other N-terminal epitope for TDP-43, the N-terminal HA tag did not generally image with acceptable contrast or signal-to-noise in paraformaldehyde-fixed stress granules. Therefore, for many of the images presented**,** we used mAb clone 3H8 against TDP-43 as indicated but confirmed transfection with the HA antibody on another fluorescent channel after using a triple-labeling technique that minimizes cross-reactivity of same-species antibodies and using anti-HA antibody before the pan-TDP-43 antibody. Specifically, triple staining was performed with mouse anti-HA.11 overnight followed by first Cy5-conjugated (1∶200 for 1 h), then unconjugated (1∶50 for 3 h) goat anti-mouse monovalent F_ab_ (Jackson Immunoresearch), followed by immunochemistry as described using mouse anti-TDP-43 clone 3H8 and one of the rabbit antibodies against hnRNP A0, G3BP, PABPC1, or eIF4A1 listed above.

Motor neurons were fixed for 15 minutes with 4 percent paraformaldehyde in PBS at 3 days *in vitro*. Mouse anti-TDP-43 (1∶1000; Abcam 3H8) and rabbit anti-G3BP (1∶100), eIF4A (1∶100), and PABPC1 (1∶30) antibodies were incubated overnight at 4°C. Cy3- or Cy2-conjugated secondary antibodies (Jackson Immunoresearch) were incubated for 1 h at room temperature. Z-series (5 to 10 sections, 0.2 µm thickness) were acquired with an epifluorescence microscope (Ti, Nikon Instruments Inc., Melville NY) equipped with a cooled CCD camera (HQ2, Photometrics, Tucson AZ). Images were deconvolved using Autoquant (MediaCybernetics, Bethesda MD) and analyzed with ImageJ.

### Western Blotting

Immunoblotting was performed according to standard procedures as reported previously [7]. Briefly, samples in Laemmli sample buffer were resolved by SDS-PAGE before an overnight transfer to Immobilon-P membranes (Millipore, Bedford MA). Blots were blocked with TBS plus blocking buffer (USB Corporation, Cleveland OH) at room temperature for 30 min and probed with primary antibodies in TBS plus 0.1% Tween-20 plus blocking buffer overnight at 4°C or for 60 minutes at room temperature. Then, blots were rinsed and incubated with secondary antibodies conjugated to fluorophores (Molecular Probes/Invitrogen, Carlsbad CA) for one hour at room temperature. Images were captured using an Odyssey Image Station (LI-COR, Lincoln NE). Blots visualizing native TDP-43 were generated using rabbit anti-TDP-43 antibody raised to recombinant TDP-43 (10782-2-AP from Proteintech Group, Chicago IL).

### Reverse Transcriptase Polymerase Chain Reaction (RT-PCR) for Detection of Endogenous TDP-S6

A primer set capable of amplifying both human TDP-43 and TDP-S6 mRNA was selected from the National Center for Biotechnology Information reference sequence NM_007375.3 for TDP-43. The forward or 5′ sense primer, 5′-ACATCCGATTTAATAGTGTTGG-3′, occurs in exon 3 while the reverse or 3′ primer in exon 6 of TDP-43, 5′-ACAGCACTACTTTCAATGAAGTG-3′, has its 3′ end 32 bp downstream of the TDP-S6 specific junction, which is 100% conserved in TDP-43 mRNA from the mouse TDP-S6 mRNA (European Nucleotide Archive sequence ACV52544.1) over 39 bases 5′ of the junction and 57 bases 3′ of the junction, with 951 intervening bases in the TDP-43 mRNA. Predicted amplicons for TDP-S6 and TDP-43 were 580 bp and 1531 bp. Total RNA was extracted from three treatment groups of HEK-293 cells (control, 0.5 mM sodium arsenite treated for 90 min, or TDP-S6 transfected for 60 h) in biological duplicate using Trizol reagent (Invitrogen) according to manufacturer’s protocol. Superscript III one step RT-PCR with Platinum Taq (Invitrogen) was used according to manufacturer’s directions with 100 ng RNA input and cDNA synthesis for 20 min at 53°C. Twenty-five percent of RT-PCR products were visualized on 3% TAE agarose.

### Stable Isotope Labeling with Amino Acids in Cell Culture (SILAC) and LC-MS/MS

HEK-293 cells were cultured in DMEM (deficient in L-Lysine and L-Arginine) supplemented with 2% dialyzed fetal calf serum (Invitrogen) as described [13]. For stable isotopic labeling, heavy forms L-Arginine (Arg10; ^13^C_6_^15^N_4_) and L-Lysine (Lys8; ^13^C_6_^15^N_2_) were added (Cambridge Isotope Laboratories, Cambridge MA) to a final concentration of 0.26 mM. Excess proline was added at 200 mg/L to block arginine to proline conversion [14]. The RIPA and urea fractions were isolated as described above. Prior to SDS-PAGE, 10 µg of heavy labeled urea fraction was added to 10 µg of unlabeled (light) urea fraction from mock, TDP-43 and TDP-S6 transfected cells. The mixed (light and heavy) urea fractions were reduced with 10 mM DTT, and resolved on a 10% polyacrylamide SDS gel. After staining with Coomassie G-250, one gel lane was cut into three gel pieces according to molecular weight (≤50, 50–100, ≥100 kDa, respectively), subjected to in-gel digestion (12.5 ng/µl trypsin). Extracted peptides were loaded onto a C_18_ column (75 µm i.d., 10 cm long, ∼300 nl/min flow rate, 3 µm resin from Michrom Bioresources, Auburn CA) and eluted during a 10–30% gradient (Buffer A: 0.4% acetic acid, 0.005% heptafluorobutyric acid, and 5% AcN; Buffer B: 0.4% acetic acid, 0.005% heptafluorobutyric acid, and 95% AcN). The eluted peptides were detected by Orbitrap (350–1500 m/z, 1,000,000 automatic gating control (AGC) target, 1,000 ms maximum ion time, resolution 60,000 FWHM) followed by five data-dependent MS/MS scans in LTQ (2 m/z isolation width, 35% collision energy, 5,000 AGC target, 150 ms maximum ion time) on a hybrid mass spectrometer (Thermo Finnigan, San Jose, CA). All data were converted from .RAW files to the .DTA format using ExtractMS version 2.0 (Thermo) and searched against the human reference database downloaded from the National Center for Biotechnology Information (downloaded July 2010) using the SEQUEST Sorcerer algorithm (version 3.11, Sage-N Research, Milpitas CA). Searching parameters included mass tolerance of precursor ions (±50 ppm) and product ion (±0.5 m/z), partial tryptic restriction, with a dynamic mass shifts for oxidized Met (+15.9949), Lys (+8.01420 for ^13^C_6_^15^N_2_) and Arg (+10.00827 for ^13^C_6_^15^N_4_), four maximal modification sites and two maximal missed cleavages. Only b and y ions were considered during the database match. To evaluate false discovery rate (FDR), all original protein sequences were reversed to generate a decoy database that was concatenated to the original database (77,764 entries) [15]. The FDR was estimated by the number of decoy matches (nd) and total number of assigned matches (nt). FDR = 2*nd/nt, assuming mismatches in the original database were the same as in the decoy database. To remove false positive matches, assigned peptides were grouped by a combination of trypticity (fully and partial) and precursor ion-charge state (2+, 3+, and 4+). Each group was first filtered by mass accuracy (15 ppm for high-resolution MS), and by dynamically increasing correlation coefficient (x_corr_, minimum 1.0) and ΔCn (minimum 0.05) values to reduce protein FDR to less than 1 percent. All MS/MS spectra for proteins identified by a single peptide and those modified by ubiquitin (lysine +114.0429) or methylation (lysine or arginine mono: +14.0156, di: +28.0313) were manually inspected as described previously [16]. Using a second search algorithm (Mascot), 58 of 59 SEQUEST database match results for peptides with PTMs were confirmed by re-searching individual.DTA files on the Matrix Science (Boston, MA) website (www.matrixscience.com; May 20, 2011) against the full human database using variable modification settings for the PTMs, ±20 ppm peptide (with up to one ^13^C atom allowed) and ±0.6 Da MS/MS tolerances. Importantly, Mascot was able to discriminate multiple site modified peptides with mixed modifications, e.g. monomethylation and dimethylation on a single peptide. The identified proteins/peptides are listed in Tables S1, S2, S3, 4, and S5. If peptides were matched to multiple members of a protein family, the matched members were clustered into a single group. Quantitative pair-wise comparisons of control, TDP-S6 and TDP-43 transfected cells were carried out according to a reported method [7]. All peptides with extracted ion intensity (signal-to-noise) are provided in Table S2, and all post-translationally modified peptides identified are listed in Table S4.

**Table 2:** Functional annotation of enriched proteins in TDP-43 or TDP-S6 detergent insoluble fraction of HEK-293 cells.

												Log_2_ Insoluble Enrichment ±SD				
Functional Annotation			p-Value	Proteins								TDP-43/Mock		TDP-S6/Mock		
DDX17DDX39ADDX39BDDX5HNRNPA0HNRNPH1			HNRNPH3HNRNPKHNRNPLHNRNPMHNRNPUHNRPDL		KHDRBS1NPM1PABPC1PABPC4PABPN1PRPF19				PTBP1RBM39RPL5RPL7RPL14RPS6			SF3A3SF3B3SFPQSFRS1SFRS13ASFRS3			SFRS6SFRS7SFRS9SSBTARDBPU2AF1	
**biosynthesis of protein**			**3.03×10^−13^**	**22**								**0.71±0.11**		**0.56±0.38**		
CALRDHX9EEF1A1EEF1A2	EEF2EIF4A1EIF4A2EIF4A3				GNB2L1HNRNPKHSPA5IGF2BP1				NACANCLPABPC1PABPC4			PPP1CAPTBP1RPS3ARPS6			SSBTUFM	
**translation**			**3.75×10^−13^**	**21**								**0.71±0.11**		**0.53±0.36**		
CALRDHX9EEF1A1EEF1A2	EEF2 EIF4A1 EIF4A2 EIF4A3				GNB2L1HNRNPKHSPA5IGF2BP1				NACANCLPABPC4PPP1CA			PTBP1RPS3ARPS6SSB			TUFM	
**splicing of RNA**			**1.00×10^−12^**	**15**								**0.89±0.73**		**0.75±1.55**		
DDX39ADDX39BHNRNPH1	HNRNPH3HNRNPMNPM1				PRPF19PTBP1SFPQ				SFRS1SFRS13ASFRS3			SFRS6SFRS7SFRS9			TARDBP	
**synthesis of protein**			**3.86×10^−12^**	**27**								**0.70±0.14**		**0.52±0.39**		
CALRDHX9EEF1A1EEF1A2EEF2	EIF3AEIF4A1EIF4A2EIF4A3EWSR1				GNB2L1HNRNPKHSPA5IGF2BP1KHDRBS1				NACANCLNPM1PABPC1PABPC4			PPP1CAPTBP1RPS3ARPS6SSB			STIP1TUFM	
**cell death**			**1.57×10^−10^**			**82**						**0.73±0.35**				**0.47±0.71**
ANP32AANXA2C1QBPCACYBPCALRCCT2CCT3CCT4CCT5CCT7CSE1LCYB5R3DDX17DDX3X	DDX4DDX5DHX9EEF1A1EEF1A2EEF1DEEF2ENO1EWSR1FASNFKBP4FLNAGAPDHGNB2L1				GSTP1HMGB1HSD17B10HSP90AA1HSP90AB1HSP90B1HSPA1A/1BHSPA5HSPA8HSPA9IGF2BP1KHDRBS1MCM2NCL				NPM1NUP210P4HBPA2G4PARP1PCBP2PCNAPDIA3PHBPPP1CAPPP2CAPPP2CBPRDX1PRDX3			PRDX6PRPF19PRPHRBBP4RPLP0RPS3ARPS6RUVBL2SERBP1SFNSOD1SFRS1STIP1STOML2			TARDBPUBA1VCPVIMXRCC5XRCC6YBX1YWHABYWHAEYWHAGYWHAQYWHAZ	
**Apoptosis**			**1.72×10^−09^**			**66**						**0.69±0.15**				**0.41±0.28**
ANP32AANXA2C1QBPCALRCCT2CSE1LDDX17DDX3XDDX5EEF1A2EEF1D		ENO1EWSR1FASNFKBP4GAPDHGNB2L1GSTP1HMGB1HSD17B10HSP90AA1HSP90AB1					HSP90B1HSPA1A/1BHSPA5HSPA8HSPA9IGF2BP1KHDRBS1MCM2NCLNPM1P4HB			PA2G4PARP1PCBP2PCNAPDIA3PHBPPP2CAPPP2CBPRDX1PRDX3PRDX6			PRPF19PRPHRBBP4RPLP0RPS3ARPS6RUVBL2SERBP1SFNSOD1SFRS1			STIP1STOML2VCPVIMXRCC5XRCC6YBX1YWHABYWHAEYWHAQYWHAZ
**endoplasmic reticulum** **stress response**			**3.65×10^−08^**			**10**						**0.57±0.1**				**0.44±0.2**
CALRHSPA1L		HSP90AA1HSP90AB1					HSP90B1HSPA1A/1B				HSPA5HSPA6		SERPINH1			VCP
**folding of protein**			**5.21×10^−08^**			**9**						**0.63±0.07**				**0.38±0.16**
CALRCANX		CCT4					HSP90AA1HSP90AB1				HSPA1A/1BHSPA5		HSPA8			RUVBL2

### Targeted LC-MS/MS for Quantification of Endogenous TDP-S6 and Related Peptides

Control, 0.5 mM sodium arsenite-treated (90 min), and TDP-S6 transfected (60 h) HEK-293 (5×10^5^) cells were harvested in RIPA buffer, and RIPA-insoluble proteins were solubilized in urea buffer as described above. All RIPA insoluble protein was loaded on a 10% SDS-PAGE gel, and proteins below 50 kDa were excised and digested with trypsin. Peptides were loaded and eluted off a C_18_ nanocolumn (75 µm i.d., 10 cm long, 300 nl/min flow rate) during a reverse-phase LC 10–30% gradient (Buffer A: 0.1% formic acid, and 1% AcN; Buffer B: 0.1% formic acid, and 95% AcN) were monitored and fragmented at precursor m/z and compared to previously identified MS/MS spectra for (a, F_276_GGNPGGFGNQGGFGNSR; b, F_152_TEYETQVK) two TDP-43 peptides (one, b, shared with TDP-S6), (c, F_276_GVHLISNVYGR) one peptide unique to TDP-S6, (d, D_194_QIYDIFQK) an eIF4A1 peptide unique to both eIF4A1 splicing isoforms, and (e, H_(101 or 138)_VFGESDELIGQK; f, V_(161 or 198)_VLAYEPVWAIGTGK) two triosephosphate isomerase peptides found to be constitutively present at high levels in the detergent insoluble fraction with no significant effect of TDP-S6 overexpression on quantitation in this fraction, and thus suitable for normalization. MS/MS settings were: 1.7 m/z isolation width centered at [(a) 573.28, (b) 864.39, (c) 681.87, (d) 585.80, (e) 730.37, (f) 802.45], 35% collision energy, 5,000 AGC target, 150 ms maximum ion time. Each sample was run in technical replicate.

Thermo .RAW files were analyzed manually using Qual Browser 2.0.7 (Thermo). MS/MS signal intensity was summed for the two or three most prominent fragment ions from each of the TDP peptides a-c [a1 993.45 (y_10_), a2 1351.61 (y_14_); b1 896.44 (y_7_), b2 767.39 (y_6_), b3 604.33 (y_5_); c1 808.43 (y_7_), c2 695.35 (y_6_), c3 921.51 (y_8_)], and total summed intensity for each peptide was multiplied by 150 ms/(ion inject time) to cancel out effects of AGC on MS2 fragment ion intensities, particularly in weak and multiplexed MS/MS spectra. All fragment ions used for quantification were required to be present for a positive identification and accurate quantification. Extracted ion current (XIC) intensity of the precursor ion not subject to AGC was used to quantify eIF4A1 and triosephosphate isomerase peptides. Each of the triosephosphate isomerase peptide intensities was then used to normalize each TDP or eIF4A1 peptide intensity before calculation of arsenite-treated/control or TDP-S6 transfected/control relative intensity. Finally, the two triosephosphate isomerase peptide-normalized intensities for each of the other peptides were averaged and presented as log_2_(experimental/control) ±95% confidence interval.

### Structural Alignment

Protein data bank nuclear magnetic resonance-derived structures of human TDP-43 RRM1 (PDB ID: 2CQG) and RRM2 with bound TAR ssDNA [17] were aligned in Swiss-PDB Viewer 4.0.1 (Swiss Institute of Bioinformatics) using the magic fit function, resulting in an α-carbon RMSD of 1.12 Å (calculated for 64 atoms). RRM2 amino acids were masked from view, and figure images were exported and rendered in POV-Ray 3.7.b38.

### External Reference Standard Peptides for TDP-43 Ubiquitination

IAA modification (dialkylation resulting in a 2-acetamidoacetamide adduct) of lysine residues in proteins at elevated temperature prior to trypsin digestion results in a peptide which is mass-identical to the same peptide modified at the same lysine residue by ubiquitin [18]; i.e., tryptic digestion of such ubiquitin-linked isopeptides results in a glycine-glycine tagged lysine residue with the same atomic composition as lysine epsilon-amino-diacetamide. pGS21-TDP-43 expressing GST-TDP-43 was transformed into the *E. coli* BL21 strain and a monoclonal culture was expanded in 150 mL TB media until reaching an OD_600_ of 0.6 and induced for 6 h with 0.1 M IPTG (Sigma, St. Louis MO) at 28°C. Isolation of GST-TDP-43 and binding to glutathione sepharose (Amersham, Piscataway NJ) was carried out according to the protocol of Frangioni and Neel [19]. Protein on beads was eluted during alkylation in 10 mM IAA (Sigma, St. Louis MO) for 10 min at 85°C, run in an SDS-polyacrylamide gel, Coomassie stained for protein weight estimation (5 µg of protein banding at 70 kDa), trypsin digested and gel extracted prior to LC-MS/MS and database search with dynamic modification of +114.0429 Da on lysine and up to two missed cleavages. These precursor charge states were then used to establish a targeted MS/MS method for detection of mass-identical true lysine-GG peptides. Segments were defined to increase the robustness of the duty cycle at any given time, i.e. reduce the number of peptides monitored for multiple charge states at any given time. A similar LC gradient to the one used for discovery extending over 60 minutes was employed. Spectra for the true lysine-GG peptides were confirmed by comparison to the database-searched pseudo-GG peptide MS/MS spectra, and by similar relative elution times.

### Mutagenesis of TDP-43 and TDP-S6 Plasmids

The Stratagene Quikchange II mutagenesis kit (Agilent, Santa Clara CA) in combination with PAGE-purified site-specific primers (IDT, Coralville IA) was used to generate pCDNA3.1 plasmids encoding HA-tagged TDP-43 with point mutations introducing lysine to arginine substitutions at K102, K114, K145, and/or K181. All mutants were confirmed by DNA sequencing.

**Table 3:** Proteins consistently elevated in TDP-S6 detergent insoluble fraction relative to TDP-43.

		Log_2_ Insoluble Enrichment ±SD	
A. Proteins from Annotated Functional Categories in Table 2		TDP-43/Mock	TDP-S6/Mock
***EIF4A1***	***eukaryotic translation initiation factor 4A, isoform 1***	0.81	1.03
***EIF4A2***	***eukaryotic translation initiation factor 4A, isoform 2***	0.88	1.09
***GNB2L1***	***RACK1; guanine nucleotide binding protein (G protein), beta 2-like 1***	0.72	0.96
**HSPA1L**	***heat shock 70 kDa protein 1-like***	0.35	0.73
***IGF2BP1***	***ZBP1; insulin-like growth factor 2 mRNA binding protein 1***	0.68	0.82
***KHDRBS1***	***Sam68; KH domain containing, RNA binding, signal transduction associated 1***	0.41	0.99
***PA2G4***	***EBP1; proliferation-associated 2G4, 38 kDa***	0.67	0.77
***PABPC1***	***poly(A) binding protein, cytoplasmic 1***	0.76	1.25
***PABPC4***	***poly(A) binding protein, cytoplasmic 4 (inducible form)***	0.87	1.46
***PARP1***	***poly (ADP-ribose) polymerase family, member 1***	0.54	0.72
PRDX3	peroxiredoxin 3	0.98	1.34
***TARDBP***	***TDP-43; TAR DNA binding protein***	3.51	6.32
U2AF1	U2 small nuclear RNA auxiliary factor 1	0.96	1.26
VCP	valosin-containing protein	0.53	0.69
**B. Other Proteins**			
***G3BP1***	***GTPase activating protein (SH3 domain) binding protein 1***	0.00	1.70
LONP1	lon peptidase 1, mitochondrial	0.38	0.70
PSMA4	proteasome subunit, alpha type, 4	0.38	0.71
RAB15	RAB15, member RAS onocogene family	0.00	0.71
RBMX	RNA binding motif protein, X-linked	0.47	0.80
RBMXL2	RNA binding motif protein, X-linked-like 2	0.35	0.61
TMPO	thymopoietin	0.47	0.73
UBC	ubiquitin	−0.02	1.35

Symbols for cytoplasmic stress granule proteins or proteins involved in translational repression are indicated by bold, italicized text.

## Results and Discussion

### Quantitative Proteomics of TDP-43 and TDP-S6 Expressing Cells

Over-expressed human TDP-43 and aggregate prone TDP-S6 with N-terminal HA tags were previously characterized [7]. Over-expressed TDP-43 has primarily nuclear localization, whereas TDP-S6 forms highly insoluble aggregates primarily localized to often large (>10 µm) cytoplasmic structures and smaller (<2 µm) nuclear structures (**Figure 1A**). Methods for enrichment such as immunoprecipitation require a balance in conditions, permitting solubilization of protein by detergent or weak chaotropes, but maintaining non-covalent interactions within intact protein complexes. In contrast, for this study, proteins potentially co-aggregating with overexpressed TDP-43 or TDP-S6 were enriched and separated from potential contaminants, which were removed from the isolated aggregates by detergent-rich (RIPA) buffer extraction. The detergent insoluble aggregates were then solubilized in buffer containing 8 M urea and mixed with stable isotope-labeled internal standard proteins after fractionation (a strategy to preserve detection capacity for dynamic interactions [20]). This preceded quantification by unbiased mass spectrometry to identify and rank both covalent and non-covalent partners associated with the detergent insoluble fraction including detergent insoluble aggregates of TDP-43 or TDP-S6.

Detergent insoluble fractions were prepared from untransfected control stable isotope-labeled (SILAC) HEK-293 cells and three pools of transfected HEK-293 cells [vector (Mock), TDP-43 and TDP-S6]. Consistent with previous results following sarkosyl-detergent extraction [7], TDP-S6 was highly (>95%) detergent insoluble (**Figure 1B**). Each of the three transfected insoluble fractions were subsequently mixed with equal protein weight of untransfected internal standard (detergent insoluble in replicate 1 or soluble in replicate 2) and characterized by LC-MS/MS followed by peptide identification and quantitation (**Figure 1C**). This differs from the multiplex SILAC approach used previously [7] in which mixing cells prior to detergent extraction largely eliminates experimental variability, but potentially masks dynamic, non-covalent, interactions [20]. Also in contrast to the multiplex approach, the current method employs the two heavy protein fractions only as pools of internal standards for quantification of native peptides and proteins in the detergent insoluble fraction from transfected cells. This experimental approach, performing two replicates, with heavy labeled internal standards from either the detergent soluble or insoluble fraction, and analyzing only two fractional proteomes at a time, reduces the complexity of each sample in the mass spectrometer, thus enhancing sampling coverage of the quantified proteome.

**Figure 3:**
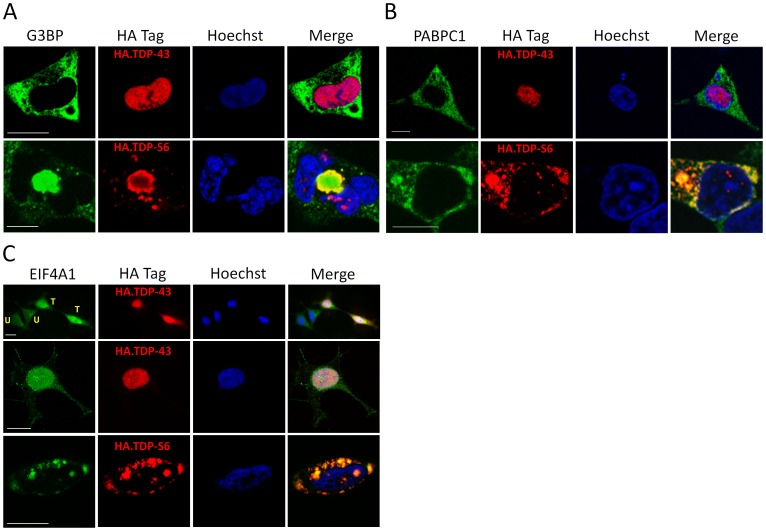
Subcellular localization of stress granule proteins coaggregating with TDP-S6 in HEK-293 cells. Immunofluorecence confocal microscopy analysis of overexpressed HA.TDP-43 or HA.TDP-S6 (red) with endogenous G3BP (**A**), PABPC1 (**B**), or eIF4A1 (**C**) (green) in HEK-293 cells. Hoechst stain for the nucleus is shown in blue. In panel C, “T” indicates a transfected cell and “U,” untransfected. Scale bars, 10 µm.

### Identification of Detergent Insoluble Proteins Significantly Changing in Response to TDP-43 and TDP-S6 Overexpression

Each protein mixture of internal standard and the detergent insoluble extract of one of the three transfected cell populations was resolved on an SDS-PAGE gel, excised into three molecular weight regions, and digested into tryptic peptides. Then each sample was analyzed by LC-MS/MS on a high-resolution Orbitrap mass spectrometer. Log_2_-transformed (native/internal standard) ratio for proteins quantified in one or more molecular weight regions of both replicates (soluble and detergent insoluble internal standard) was calculated using IQUAN software [21] and produced a list of 585 proteins quantified in the detergent insoluble fraction of all three transfected cell populations (mock, TDP-43, and TDP-S6) in both replicates (**Table S1**). The quantified protein populations showed a Gaussian distribution in mock transfected cells, but an uneven or possibly bimodal distribution in the TDP-43 or TDP-S6 experiments (**Figure 1D**). These departures from the normal distribution are consistent with an effect of TDP-43 or TDP-S6 protein overexpression on the detergent insoluble proteome due to co-aggregation effects.

From the list of 585 proteins quantified, we present the subset of proteins (*n* = 41; 35 increasing and 6 decreasing) consistently changing more than 1.64 times the standard deviation of the mean (beyond the 95 percent one-tailed confidence interval, or p<0.05) in the TDP-43 and/or TDP-S6 cell models relative to mock transfected HEK-293 cells, or in the TDP-43 model relative to TDP-S6 (**Table 1**). This significance cutoff for each comparison respectively fell at ±0.91 log_2_(TDP-43/Mock), ±0.89 log_2_(TDP-S6/Mock), and ±0.65 log_2_(TDP-S6/TDP-43). To visualize proteins significantly changing in the TDP-43 and/or TDP-S6 models, we constructed a triple SILAC map [22] of the differences in log_2_-transformed ratios (**Figure 1E**). This map is shown after recentering differences by subtracting the population means. For significantly changing proteins along the y-axis, lighter orange points indicate the magnitude of change of blue points at equivalent x values in terms of the simpler TDP-S6/Mock ratio. Confirming a high burden of TDP-43 in the transfected cell models, we identified TDP-43 as the most enriched detergent insoluble protein (beyond the top right corner of the map), with an 11-fold increase relative to mock transfection in the TDP-43 model, and an 80-fold increase in the TDP-S6 model. Consistent with earlier results [7], we found SUMO2/3 enriched in TDP-43 model detergent insoluble extracts and further elevated in TDP-S6 extracts, whereas ubiquitin was specifically enriched in the TDP-S6 detergent insoluble extract.

In the first three of four groups of changing proteins in Table 1, significant changes were detected in the detergent insoluble proteomes for those proteins in either or both of the two overexpression models compared to mock transfected cells. Members of the fourth group in Table 1 are designated by the downward trending arrow (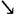) and are enriched with TDP-43 overexpression compared to a relative depletion in TDP-S6 extracts (all below −0.65 log_2_(TDP-S6/TDP-43), consistent with interactors of the TDP-43 C-terminal region that is missing in TDP-S6; some of the group 4 proteins are significantly changed by other measures in groups 1 to 3 and are not repeated, but rather are denoted above by an additional diagonal downward arrow.

Interestingly, nine out of the 35 proteins (25.7%) increasing significantly in TDP-43 and/or TDP-S6 insoluble fractions contain at least one RRM domain. Manual comparison of MS spectra for differences in light/heavy ratios in mock, TDP-43 and TDP-S6 models confirms the changes provided by automatic data analysis for four such proteins: PABPC1, G3BP, RBM14, and PSF (**Figure 2A**). Two of the RRM proteins in Figure 2A co-enriching with TDP-S6 are PABPC1 and G3BP, whereas the proteins RBM14, PTB associated splicing factor (PSF), and NonO co-enriched with TDP-43 and/or TDP-S6. We validated this pattern of enrichment in the insoluble fraction of biological replicates from mock, TDP-43, and TDP-S6 transfected HEK-293 cells by western blot (**Figure 2B**). These results are consistent with our proteomics, indicating selective enrichment of G3BP and PABP1 in the TDP-S6 detergent insoluble proteome, and less selective increases in PSF and NonO with either TDP-43 or TDP-S6 overexpression.

Two recent publications identified full length TDP-43 interaction partners following overexpression and immunoprecipitation from HEK-293 lysates [23] and stable expression and tandem affinity purification from HeLa lysates [24]. Comparison of the interaction partners identified by these studies with the proteins in Table 1 that specifically increase in TDP-43 and/or TDP-S6 insoluble extracts (**Table 1**, right column) indicate that 60 percent (21 of 35 increasing proteins) overlap with the reported TDP-43 cell model interactomes. It is reasonable to conclude that detergent extraction of soluble proteins from cell model lysates provides sufficient enrichment of TDP-43 or TDP-S6 to enable identification of TDP-43 interaction partners in the detergent insoluble fraction of these cells (i.e. the 35 increasing proteins are candidates for coaggregation with TDP-43 and/or TDP-S6).

In this context, the data set is useful in generating hypotheses relating to functional crosstalk in discrete compartments or complexes that harbor the coenriched proteins. For example, a number of RNA binding proteins known to localize to nuclear substructures active in splicing function (RBM14, PSF, NonO and others) are coenriched with TDP-43 and/or TDP-S6. In addition, cytoplasmic RNA binding proteins which participate in cytoplasmic stress granules that have a role in stress-induced translational repression of housekeeping gene mRNAs [G3BP, PABPC1, eIF4A1, KHDRBS1/Sam68, and GNB2L1/RACK1 (non-RNA binding)] also coenriched. This raises the question if overexpressed TDP-43 or TDP-S6 localize in either paraspeckles or stress granules, which is addressed in below results sections.

Other novel observations discussed here (but not validated further) are that TDP-43 more than TDP-S6 preferentially accumulates with the previously reported interacting 60 S and 40 S ribosomal proteins L5, S3a, L14, P0, and L3, and the nascent polypeptide chaperone complex represented by Cct7 in group 3. Group 4 proteins that significantly decrease in the comparison of TDP-43 to TDP-S6 insoluble proteome include dynein and EWS (in both groups 3 and 4), as well as RNA helicase A. The latter two proteins interact in a drug-sensitive nuclear complex [25]. Dynein enrichment along with ribosomes accumulating with TDP-43 could indicate TDP-43 participation (more than TDP-S6) in RNA transport granule assemblies associating with ribosomes (since both 60 S and 40 S subunits were enriched) [26]. Translation function of the ribosome acting on certain mRNAs would be further enabled by helicase A unwinding of tertiary RNA structure [27]. Thus TDP-43, more tightly than TDP-S6, may link nuclear target mRNA transcription, splicing, and translation via intact dynein-dependent mRNA transport function. Conversely, TDP-S6 has higher co-enrichment with select cytoplasmic factors independent of elongating core ribosome assemblies that have been shown to strongly influence ribosome function, including translation initiation factor 4A (eIF4A1) and guanine nucleotide-binding protein ß2-like 1 (GNB2L1/RACK1) [28] in group 2. Generalizing that the members of groups 3 and 4 trend down in the comparison of TDP-43 to the TDP-S6 model, it may be that these proteins’ RNA-directed functions acting on the wide array of TDP-43 associated mRNAs [29] may occur through interactions with the C-terminus of TDP-43 or via indirect interactions dependent upon an intact C-terminus not present in TDP-S6. Another discussion point regarding the decreasing detergent-resistant proteins in both the TDP-43 and TDP-S6 model including actin and myosin is that their relative absence with overexpression could be consistent with a non-stoichiometric accumulation of TDP-43 outside of functional TDP-43 complexes responsible for actin mRNA transport, translation, or stabilization normally carried out by endogenous TDP-43 in HEK-293 cells, if not also a relatively partner-independent role for TDP-43 in the degradation of actin mRNA [30], or TDP-43 concentration-dependent transcriptional repression of actin cytoskeleton components.

Recently, TDP-43 negative, FUS-positive FTLD inclusions were found to contain EWS and TAF15 [31]. We note that these 3 related proteins FUS, EWS, and TAF15 were among the 585 reliably quantified proteins in this study, but our results pose the interesting implication that overexpressed TDP-43 preferentially could co-aggregate in HEK-293 cells with EWS [0.99 log_2_(TDP-43/Mock)] rather than FUS (0.34) or TAF15 (0.37) (**Table S1**), consistent with cell type specific interactions, which may not occur in the neurons affected in either FTLD or ALS. An equally plausible alternative to the interpretation of protein-protein interaction is that TDP-43 overexpression may indirectly and differentially affect transcription, translation, or post-translational stability of these intrinsically detergent-resistant proteins that have potential overlapping function with TDP-43.

**Figure 4:**
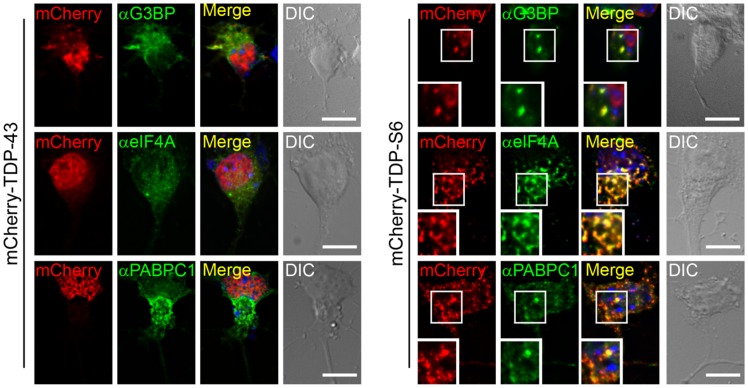
Subcellular localization of stress granule proteins coaggregating with TDP-S6 in primary motor neurons. Left panel shows localization of G3BP, eIF4A, and PABPC1 (green) in mCherry-TDP-43 overexpressing mouse motor neurons. Right panel, colocalization of the same stress granule markers with mCherry-TDP-S6 in neuronal soma. Nuclei are stained with Hoechst dye (blue). Scale bars, 10 µm.

### Two Stipulations Regarding the Quantification of a Target Aggregate Proteome

The quantitative approach employed for this study following qualitative detergent insoluble protein enrichment has two important caveats that must be addressed. First, qualitatively, the aggregate proteome is not the entire detergent insoluble proteome. In fact, the makeup of the mock-transfected detergent insoluble proteome provides insight into a background level of proteins that copurify with aggregate proteins. These proteins fall into categories of possibly ordered protein complexes with nucleic acid chains (RNPs and chromatin), cytoskeletal proteins, or mitochondrial organelle contaminants (**Figure S1**), where categories for localization, domain content, or nucleic acid association were determined using the Database for Annotation, Visualization, and Integrated Discovery (DAVID) [32]. The target aggregate proteome is best defined as the proteins which change quantitatively from the background under conditions that are expected to alter (in this case, to promote) intermolecular interactions. Under such conditions, distinct protein species significantly shift into or from the detergent (solvent) insoluble fraction, as when TDP-43 intracellular concentration is increased by overexpression. After cell lysis, solvent makeup, including salts and a number of additional stabilizers, will affect the fractionation of proteins by changing the balance between pro-aggregation intermolecular interactions and pro-solvation intramolecular folding [33]. The stringent RIPA buffer used here and an absence of freezing the transfected cell pellets before fractionation would be expected to maintain the biochemical segregation of proteins that already occurred in the live cells, by keeping folded proteins in the detergent-extracted fraction. In fact, the control (SILAC-labeled) detergent soluble and insoluble fractions (used for the two replicates strictly as internal standards) were derived from cells which had been frozen before fractionation. Freeze-thaw induced aggregation of distinct species is one explanation for differences in the relative abundance of some detergent-insoluble proteins between the SILAC (control) internal standard and mock-transfected group; increased passage number to accommodate complete labeling is another possible source of differences. Regardless, the mock transfected/control distribution of relative protein abundances in the insoluble fraction remains a normal distribution with the population mean centered at zero, representing no gross change (**Figure 1D**).

The second, quantitative, aspect of this study design requires careful consideration of mass balance. Although SILAC internal standard and analyte detergent insoluble proteomes were equally loaded by protein weight, a remaining concern is whether the light and heavy detergent insoluble protein pools are directly comparable, since the condition promoting aggregation (TDP-43 overexpression) could expand or contract the total protein amount that is resistant to detergent extraction. This issue of mass balance underpins whether any quantitation as calculated is reliably indicating the specified change in protein. In the case of TDP-43 overexpression, mass balance is roughly maintained because the total protein in the detergent insoluble fraction containing aggregates does not grossly change as a percentage (about 4 percent) of total cellular protein in lysates of TDP-43 or TDP-S6 transfected vs. untransfected HEK-293 cells [7]. However, if this experimental workflow is adapted for use in the case of another protein that more grossly alters bulk protein biochemistry, then mass balance would not be maintained and additional normalization would be necessary to provide accurate quantitation. In such a case, the number of cells from which protein is fractionated could be strictly controlled rather than the mixed protein amount, so that the target aggregate proteome amount would be allowed to reflect expansion or contraction. When protein quantity is properly controlled prior to mixing with internal standard, it should hold true that if there are *bona fide* aggregate proteins that do not change relative to the mock transfected detergent insoluble proteome, then they lie outside the “target aggregate proteome” of interest. This does not preclude the presence of aggregates in the background detergent-insoluble proteome of mock-transfected cells, but they are not identified by the method employed, because they present no quantitative change.

**Figure 5:**
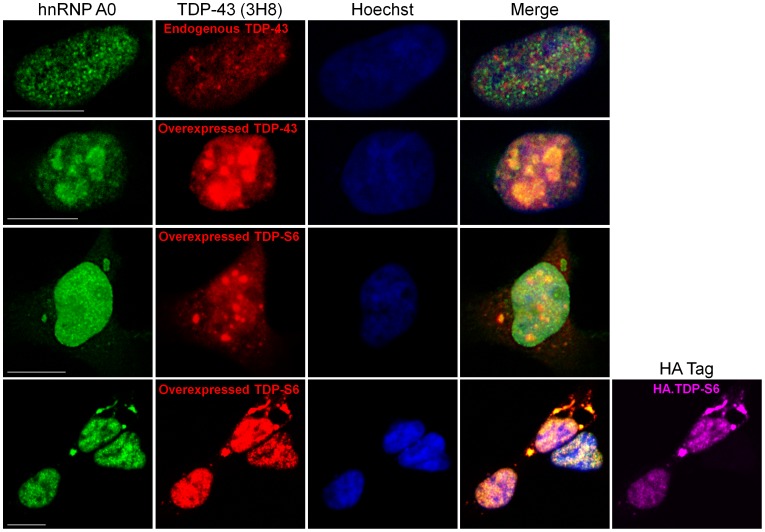
hnRNP A0 colocalization in inclusion bodies positive for overexpressed TDP-43 and TDP-S6 in HEK-293 cells. Endogenous or overexpressed TDP-43 or TDP-S6 (red) localization relative to endogenous hnRNP A0 (green). In the lower set of images, triple staining was performed to differentiate overexpressed TDP-S6 from endogenous TDP-43 in a neighboring cell; HA staining not included in the merged image is shown in magenta. Nuclei are stained with Hoechst dye (blue). Scale bars, 10 µm.

### Pathway Analysis of a Broader Group of TDP-coenriched Detergent Insoluble Proteins Confirms a Strong TDP-43 Association with Translation and TDP-S6 with Cytoplasmic Stress Granule Components

Identifications of 299 proteins increasing moderately or better, at least 0.5 log2-ratio units (41 percent) in either the TDP-43 or TDP-S6 aggregate proteome, were input into Ingenuity Pathway Analysis. Selected output functional annotation categories for these enriched proteins are given in **Table 2**, and include a general RNA processing category, translation, splicing, and cell death or apoptosis. These categories are consistent with established roles of TDP-43, and further implicate protein biosynthesis or translation, as reported in the soluble TDP-43 interactome [23]. Proteins associated with cell death or apoptosis increasing in the aggregate proteome with TDP-43 overexpression are also consistent with evidence that overexpression of wild type, full length TDP-43 leads to neuronal toxicity in particular [34], [35]. However, nuclear fragmentation was not apparent in the vast majority (>95 percent) of HEK-293 cells transfected up to 72 h with either TDP-43 or TDP-S6 by immunocytochemistry (data not shown), so it is not clear whether a shift in these proteins to the aggregate proteome represents a decrease in cellular survival mechanisms that progresses to apoptosis. The TDP-S6 model, which forms the largest TDP-nucleated or -associated aggregates, has less of an increase in detergent-insoluble death-associated proteins than the TDP-43 model, consistent with a possible protective effect.

In fact, for all of the functional categories in **Table 2**, we noted that TDP-S6 aggregate proteins were less enriched than with TDP-43 overexpression, despite the fact that TDP-specific peptides were more enriched in the TDP-S6 model. We therefore asked what the identities are of the proteins that are at least moderately enriched and are more enriched in the TDP-S6 model. Surprisingly, this short list of 22 proteins (**Table 3**) is populated by no fewer than ten proteins known to participate in cytoplasmic stress granules [36], [37]. An eleventh protein, PA2G4/EBP1, similar to eIF4A and GNB2L1/RACK1 in Table 1 group 2, has a role in modulating translation initiation complex assembly, in this case via inhibition of eIF2α phosphorylation [38], posing the possibility that overexpressed TDP-S6 interacts with PA2G4 and could alter kinase signaling that accelerates assembly of eIF2-RNA binding-ribosome preinitiation complexes, thereby modulating translation rate for some mRNAs, while cytoplasmic stress granule assembly in response to many stresses is also strongly influenced by eIF2α phosphorylation [39]. Consistent with eIF2 complex absence from stress granule-associated preinitiation complexes [39], the three eIF2 complex members were quantified by multiple peptide ratios in the second replicate with spiked-in RIPA-soluble standard, with no increase evident in the TDP-S6 model aggregate proteome (data not shown). This is in contrast to increasing eIF4A1 (**Table 1**∶1.03 TDP-S6/mock log_2_ ratio), EIF4G1 (replicate 2∶1.60 TDP-S6/mock log_2_ ratio), and EIF4H (replicate 2∶0.73 TDP-S6/mock log_2_ ratio).

**Figure 6:**
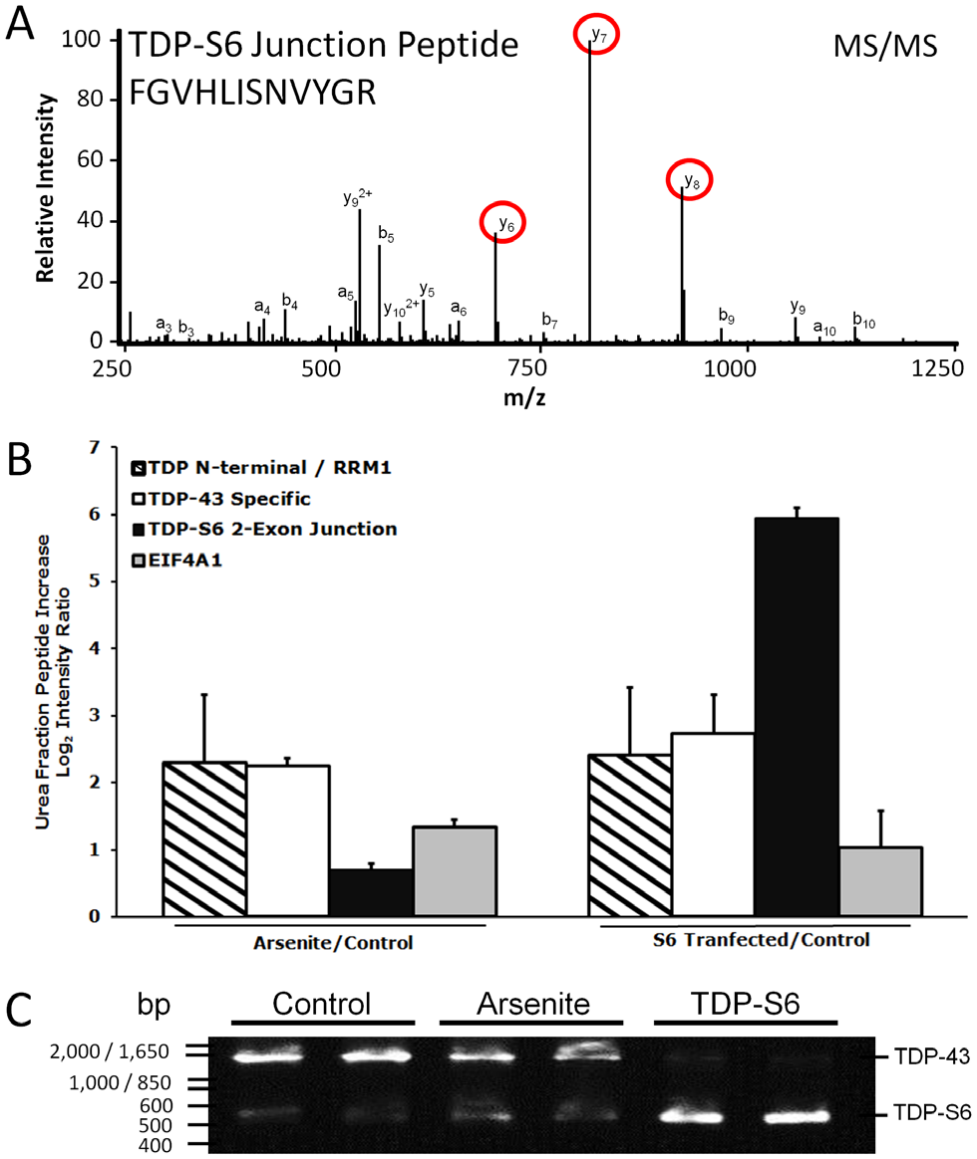
Quantification of detergent insoluble TDP-S6 and TDP-43 following arsenite treatment via LC-MS/MS monitoring of the alternative splicing exon-junction specific TDP-S6 peptide and RT-PCR determination of splicing preference in arsenite-treated HEK-293 cells. (**A**) A representative MS/MS fragmentation spectrum of TDP-S6 exon junction peptide F_276_GVHLISNVYGR, where FG is encoded by the last two codons of a shortened exon 6 in TDP-S6 and the remainder is encoded by an alternative exon composed of bases usually found in the 3′ UTR of TDP-43. The three most intense MS/MS fragment ions on our platform, y_6_, y_7_ and y_8_ are circled in red. (**B**) The summed intensity of the ions circled in (A) was used to quantify TDP-S6 as described in the methods for Targeted LC-MS/MS. Similarly, a TDP-43 specific peptide (F_276_GGNPGGFGNQGGFGNSR), and a shared RRM1 peptide F_152_TEYETQVK were quantified in arsenite-treated HEK-293 detergent insoluble fraction relative to untransfected, untreated (control) cells. Quantification of the same peptides from the detergent insoluble fraction of TDP-S6 transfected cells were measured as a positive control. EIF4A1 was also monitored (via peptide D_194_QIYDIFQK) to show co-enrichment of a stress granule marker with arsenite treatment or TDP-S6 transfection. Error bars show the 95 percent confidence interval for two replicates. Arsenite treatment (0.5 mM) was for 90 min. (**C**) Reverse transcriptase (RT)-PCR of TDP-43 and TDP-S6 mRNA species. RT-PCR was performed on total mRNA extracted from biological replicates from control or 0.5 mM arsenite-treated (90 min) HEK-293 cells. Primers selected gave predicted TDP-S6 and TDP-43 specific amplicons of 580 and 1531 bp, respectively. RNA from TDP-S6 transfected cells was used as a positive control.

**Figure 7:**
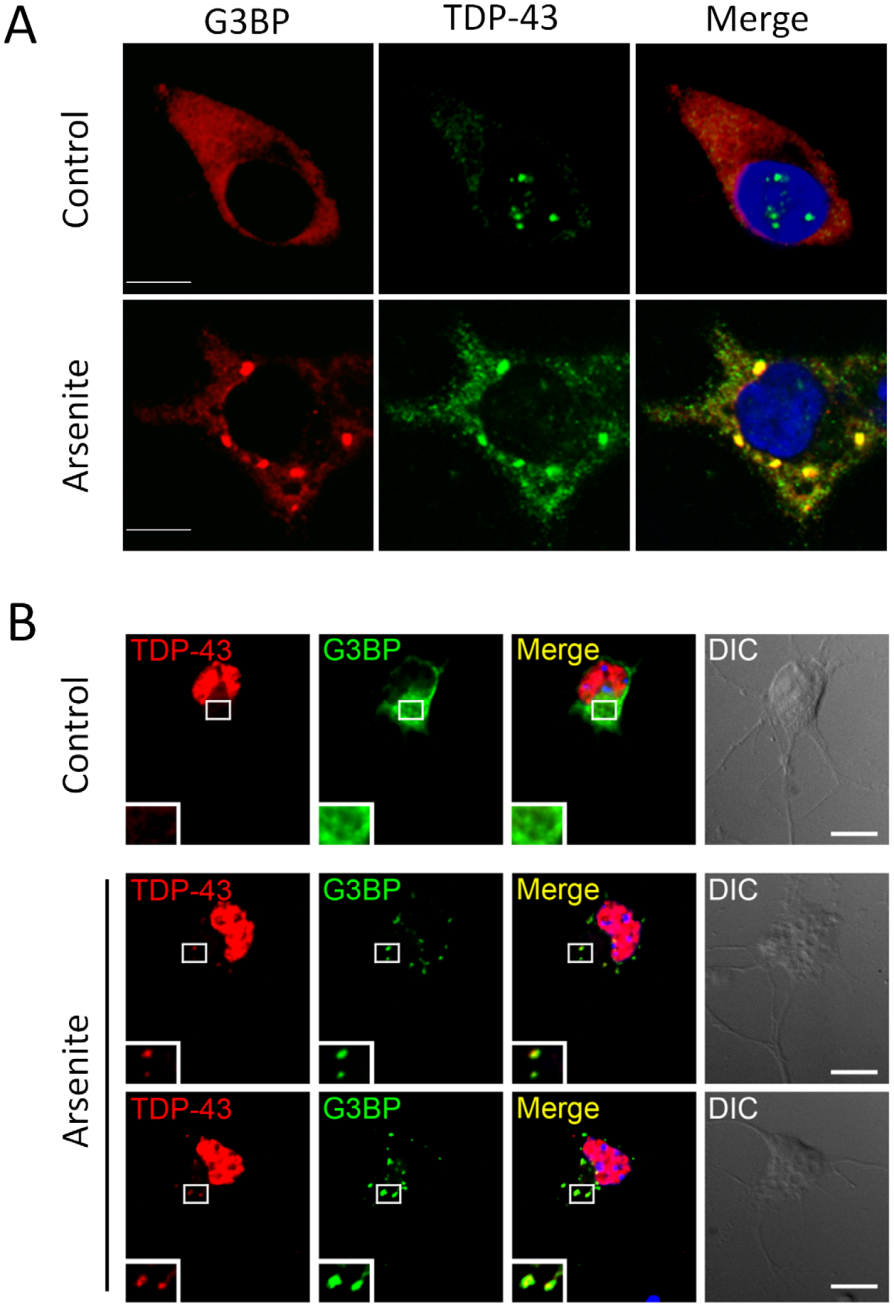
Endogenous TDP-43 participates in arsenite-induced G3BP positive stress granules. (**A**) Endogenous TDP-43 (green) stained with an N-terminal monoclonal antibody raised against residues 1–260, and G3BP (red) in control and sodium arsenite (0.5 mM, 45 min)-treated HEK-293 cells. (**B**) A small fraction of endogenous TDP-43 (red) localizes to G3BP positive (green) cytoplasmic granules induced by sodium arsenite in motor neurons. Nuclei are shown in blue. Scale bars, 10 µm.

### TDP-S6 Overexpression-associated Inclusion Bodies are Composed of Common Stress Granule Proteins in HEK-293 Cells and in Cultured Primary Mouse Motor Neurons

With a preponderance of proteomic evidence indicating a role for TDP-S6 in altering translation initiation complexes, causing both core components and modulators to become increasingly resistant to detergent solubilization, we asked whether TDP-S6-induced inclusion bodies contain stress granule components such as G3BP, PABPC1, and eIF4A1, and if any colocalization with full length HA tagged TDP-43 could be discerned. Immunocytochemistry indicated that TDP-S6 inclusions indeed are strongly positive for G3BP (**Figure 3A**), PABPC1 (**Figure 3B**), and eIF4A1 (**Figure 3C**), with apparent sequestration from an otherwise diffuse cytoplasmic distribution. Full length TDP-43 overexpression did not alter the cytoplasmic localization or patterning of G3BP or PABPC1, although eIF4A1 became increasingly nuclear with TDP-43 overexpression (**Figure 3A, 3B, 3C**). Thus, TDP-S6 inclusion bodies resemble cytoplasmic stress granules in composition of the tested components, even though they are unconventionally large in size in some cases.

To extend these findings from the proteomics and immunocytochemistry to a model relevant to neuronal proteinopathy, we performed immunocytochemistry on primary mouse motor neurons for the same endogenous stress granule markers in the presence of overexpressed fluorescent-tagged TDP-43 or TDP-S6. In the absence of any stressful insult, TDP-S6, but not TDP-43, colocalized in the cytoplasm of the neuronal soma with each of the stress granule markers (**Figure 4**). Thus, the accumulation of the TDP-S6 isoform in neurons is sufficient to nucleate or induce cytoplasmic formations that contain the stress granule markers identified in this study in the absence of any exogenous stress other than TDP-S6 overexpression.

hnRNP A0 enriched in the detergent insoluble fraction of TDP-43 and TDP-S6 more than two-fold (**Table 1**) but has not previously been associated with stress granules or TDP inclusion bodies to our knowledge. To further test the implications of aggregate proteome enrichment in our cellular models, we asked whether hnRNP A0 also participates with TDP-43 or TDP-S6 in formation of nuclear and cytoplasmic inclusion bodies. Indeed, although endogenous TDP-43 does not colocalize with hnRNP A0, grossly overexpressed full length TDP-43 reorganizes hnRNP A0 nuclear patterning and produces hnRNP A0 colocalization within nuclear inclusions; TDP-S6 overexpression draws some fraction of hnRNP A0 from its constitutive nuclear disposition into cytoplasmic TDP-S6 inclusions (**Figure 5**). We conclude that just as TDP-43 overexpression influenced a shift in eIF4A1 to the nucleus (**Figure 3C**), overexpression of either isoform of TDP remodels hnRNP A0 protein, although it is not clear if the functional RNPs of which it is a part are within these inclusions. Based on all observations of the TDP-S6 model to date, we conclude that TDP-S6 forms often large (>10 µm) cytoplasmic granular structures in cells, is conjugated to ubiquitin (possibly K63 and K48 linked polyUb chains [7]), and these cytoplasmic structures also minimally include G3BP, PABPC1, and eIF4A1. Importantly, we were able to confirm these results for the above three stress granule proteins in primary motor neurons, suggesting these observations may be relevant to neuronal degeneration in TDP-43 proteinopathies.

**Figure 8:**
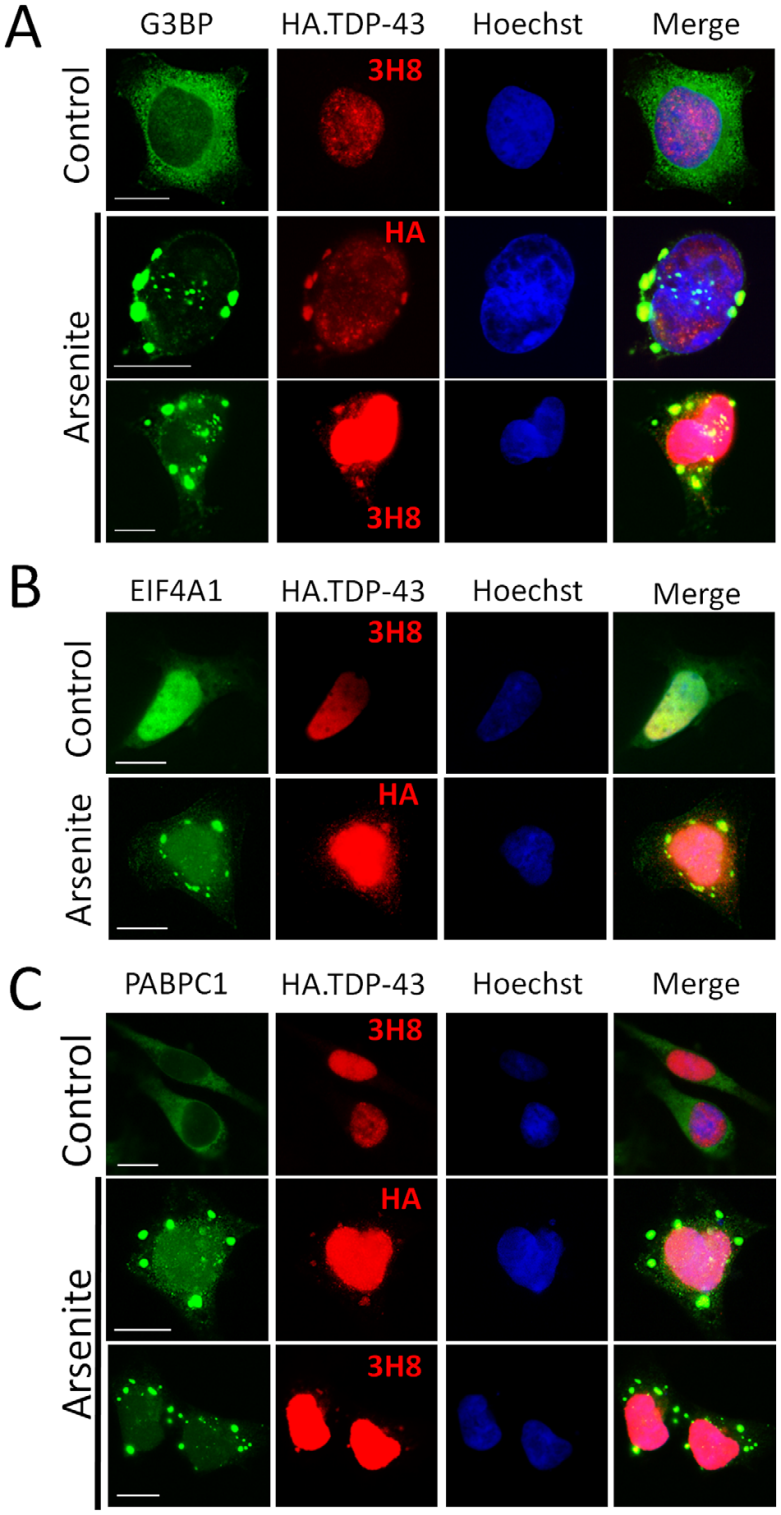
Overexpressed TDP-43 participates in arsenite-induced stress granules. (**A–C**) Overexpressed TDP-43 (red) localizes to cytoplasmic granules with redistributed G3BP (A), eIF4A1 (B), or PABPC1 (C) (green) induced by arsenite. Nuclei are shown in blue. Scale bars, 10 µm.

**Figure 9:**
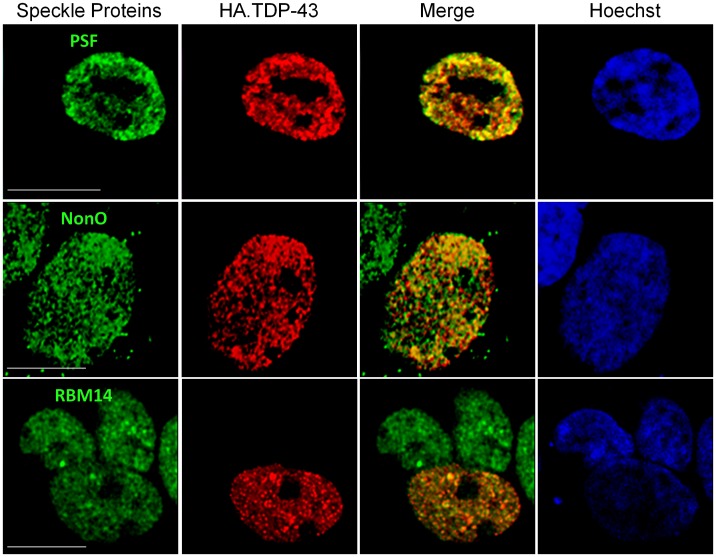
Nuclear paraspeckle proteins colocalize with TDP-43. Immunofluorecence of HA.TDP-43 (red) with endogenous target proteins (RBM14, NonO, or PSF) in green. Hoescht stain for the nucleus is shown in blue in the rightmost panels of each row. Scale bars, 10 µm.

### Endogenous TDP-43 and TDP-S6 Participate in Stress Granules, but Non-TDP-S6 Form(s) Predominate in HEK-293 Stress Granules Induced by Arsenite

A number of cellular stresses induce a dynamic convergence of mRNA transport and packaging factors at stress granules [36], [40], [41]. Recent reports have localized TDP-43 in cellular models to stress granules [23], [42]–[44] and at least one disease mutation impacts a loss of TDP-43 potentiation of stress granule formation and functional interaction with G3BP [45], whereas other familial ALS-linked mutations increase the propensity for arsenite-induced stress granules to form in a neuroblastoma cell line, and decrease detergent solubility of the protein in HEK-293 cells, which is strongly and negatively affected by arsenite treatment as well [43].

We asked if endogenous TDP-S6 might participate in arsenite-induced stress granules. Since TDP-S6 is constitutively insoluble (**Figure 1B**), we compared endogenous levels of a TDP-S6 specific peptide (**Figure 6A**) from the detergent insoluble fraction of control and arsenite-treated HEK-293 cells using targeted LC-MS/MS, with TDP-S6 transfection in a third treatment group as a positive control. Surprisingly, TDP-S6 significantly increased with 1.5 h arsenite treatment, by 62 percent [a log_2_(arsenite/control) MS/MS peak abundance ratio of 0.70±0.15; **Figure 6B**]. However, a peptide specific to other TDP-43 isoforms excluding TDP-S6 increased significantly more than the TDP-S6 specific peptide (increasing 380 percent, with a log_2_ ratio of 2.3±0.28), similar to a shared N-terminal peptide residing in both full length TDP-43 and TDP-S6 RRM1. In contrast, with TDP-S6 overexpression, the TDP-S6 peptide increased 60-fold, and the peptide that is specific for non-TDP-S6 splice isoforms increased to an extent similar to the levels seen with arsenite treatment. These results indicate that endogenous TDP-S6 may also participate in arsenite-induced detergent-insoluble cellular features along with TDP-43. The most likely candidate for such features is stress granules. One explanation for the significantly less robust increase in TDP-S6 than other TDP-43 isoforms with arsenite treatment could be the intrinsic low abundance of TDP-S6 relative to other isoforms; indeed, a rough comparison of relative abundance provided by the average raw signal intensity for each of the two exon junction peptides suggests a stoichiometry of 20∶3 for endogenous TDP-43:TDP-S6, even in the arsenite treated insoluble fraction. Moreover, no increase in mature TDP-S6 splicing product is evident via reverse transcriptase PCR of RNA from 1.5 h arsenite-treated HEK-293 cells relative to controls, although the mRNA for full length TDP-43 appears to decrease in abundance (**Figure 6C**), either due to increased splicing of other isoforms, a decrease in transcription from the TARDBP locus, or destabilization of existing TDP-43 mRNA, any of which might promote the later resolution of stress granules, since TDP-43 stabilizes them [45]. We conclude that there is a possibility that any increase in total TDP-S6 due to arsenite could be a result of enhanced translation or stability of existing TDP-S6 mRNA. However, TDP-S6 could only be measured in the detergent-insoluble fraction, and TDP-S6 contains all residues necessary for cytoplasmic aggregation of TDP-43 via intermolecular cysteine disulfide formation in response to oxidative conditions like those promoted by arsenite [46], so we cannot completely rule out that the already highly insoluble TDP-S6 has further shifted into this fraction as a result of oxidative polymerization.

The other noteworthy finding in **Figure 6B** (white bar in S6 Transfected/Control comparison) is that non-TDP-S6, TDP-43 protein participation in the detergent insoluble fraction of TDP-S6 overexpressing HEK-293 cells as measured by the exon-exon junction peptide not present in TDP-S6 increases 560±31 percent over control after TDP-S6 overexpression, even though endogenous TDP-43 transcription or mRNA stability is strongly downregulated following TDP-S6 overexpression (**Figure 6C**). We cannot definitively resolve this paradox within the scope of this study, but it is tempting to speculate that these results are consistent with (a) TDP-S6 induced isoform switching to another TDP-43 splice variant with the same non-S6 exon-exon junction peptide as full length TDP-43, but without region(s) complimentary to our primers, or (b) a drastic increase in translation efficiency, protein stability and/or aggregation propensity of endogenous TDP-43 in the presence of overexpressed TDP-S6, among other possibilities.

**Figure 10:**
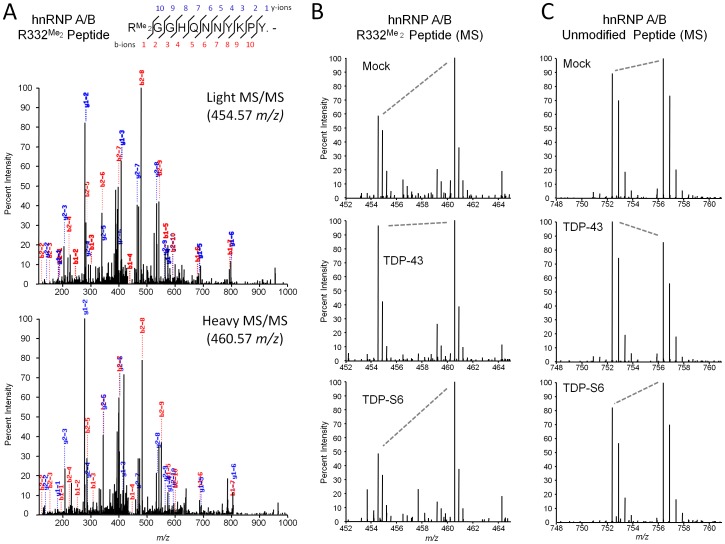
A methylated RGG motif peptide increasing with TDP-43 overexpression identified by unlabeled/SILAC peptide comparison. (**A**) MS/MS of an hnRNP A/B dimethylated R332 peptide, both unlabeled (upper panel) and heavy (lower panel). (**B**) MS spectrum for the peptide in (A) at the LC elution peak from which ion intensities for light and heavy peptides were extracted to calculate Table 4 unnormalized quantified relative levels of the modification in mock, TDP-43, and TDP-S6 paired experiments. Replicate 1 (urea/urea mixture) spectra are shown. (**C**) A representative unmodified peptide for hnRNP A/B (IFVGGLNPEATEEK), the quantified relative levels of which contributed to the normalization factors in Table 2.

**Figure 11:**
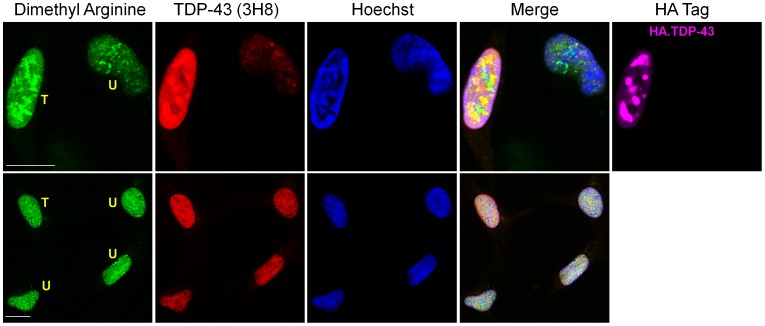
Increased asymmetric dimethylated arginine in nucleus of TDP-43 transfected HEK-293 cells. Proteins containing asymmetric dimethylarginine (green) were visualized by immunofluorescence in TDP-43 (red)-transfected and -untransfected HEK-293 cells. TDP-43 overexpression in one cell was confirmed by triple labeling also using the antibody recognizing the HA tag (upper row, rightmost panel). Nuclei are shown in blue. “T” indicates a transfected cell and “U,” untransfected. Scale bars, 10 µm.

**Table 4:** Arginine methylation site peptides identified and quantified in the TDP-43 and TDP-S6 detergent insoluble proteome of HEK-293 cells.

											*Unlabeled* *XIC Intensity/100* *(replicate 1)*			*SILAC Internal Std.* *XIC Intensity/100* *(replicate 1)*			*Light/Heavy* *Peptide Ratio*			*Modified Peptide % of Ctl (unnorm.)*		*All Unmodified* *Peptides,* *Percent of Ctl*		*Modified Peptide* *% of Control* *(Normalized)*	
Protein	Site(s)	Peptide	NCBIAccession		Charge		ExpectedMH	Ppmshift	Xcorr	MatchedIons	WT	S6	Mock	WT	S6	Mock	WT	S6	Mock	WT	S6	WT	S6	WT	S6
**hnRNP A0**	**R291-Me_2_**	R.SNSGPYR^Me2^GGYGGGGGYGGSSF.-		NP_006796.1		2	1968.8470	3.83	4.32	22/40	780	665	340	926	990	729	0.842	0.671	0.466	181%	144%	176%	172%	103%	83.9%
**Arg/Ser-rich 10 (Tra-2)**	**R241-Me_2_**	R.SYR^Me2^GGGGGGGGWR.A		NP_004584.1		3	1251.5976	7.08	3.67	25/48	961	1040	772	1610	2250	1960	0.596	0.462	0.393	152%	117%	103%	66.4%	147%	177%
**FUS/TLS**	**R216-Me_2_, R218-Me_2_**	R.GGR^Me2^GR^Me2^GGSGGGGGGGGGGYNR.S		NP_004951.1		3	1762.8439	4.86	3.55	32/80	852	768	370	846	1110	534	1.01	0.689	0.692	145%	100%	95.9%	104%	152%	95.5%
**hnRNP U**	**R733-Me_2_, R739-Me_2_**	R.R^Me2^GNMPQR^Me2^GGGGGGSGGIGYPYPR.A		NP_114032.2		3	2304.1413	5.49	3.51	35/88	390	345	98.1	727	975	320	0.536	0.354	0.307	175%	115%	114%	92%	154%	126%
**hnRNP U**	**R733-Me_2_, R739-Me_2_**	R.R^Me2^GNM^Ox^PQR^Me2^GGGGGGSGGIGYPYPR.A		NP_114032.2		4	2320.1362	6.68	3.21	38/132	654	457	408	1150	1140	1440	0.567	0.400	0.284	200%	141%	114%	92%	176%	154%
**THO complex subunit 4**	**R204-Me_2_**	R.NR^Me2^GAGGFGGGGGTR.R		NP_005773.3		3	1248.6191	6.41	3.21	25/52	1750	622	838	1710	646	1430	1.03	0.962	0.588	174%	164%	135%	112%	130%	147%
**hnRNP A/B**	**R322-Me_2_**	R.R^Me2^GGHQNNYKPY.-		NP_004490.2		3	1361.6708	5.92	3.02	19/40	1720	756	781	1780	1520	1320	0.965	0.497	0.591	163%	84.1%	112%	104%	146%	81.2%
**hnRNP D0**	**R345-Me_2_**	R.R^Me2^GGHQNSYKPY.-		NP_112738.1		3	1334.6599	6.25	2.93	23/40	2380	2090	1610	2120	2220	1670	1.12	0.941	0.964	116%	97.7%	104%	87.4%	112%	112%

### G3BP, PABPC1, and eIF4A1 are also Common Components of TDP-43 Positive Stress Granules Induced by Arsenite, Even Following TDP-43 Overexpression

It remained to be tested whether the composition of stress granules formed due to acute arsenite treatment is similar to that of TDP-S6 inclusions. Endogenous TDP-43 and/or TDP-S6 is recruited to arsenite-induced G3BP positive stress granules as visualized by an N-terminal TDP-43 antibody used on methanol-fixed HEK-293 cells (**Figure 7A**). Endogenous TDP-43 colocalization with G3BP in cytoplasmic stress granules was also induced by arsenite treatment in primary mouse motor neurons (**Figure 7B**).

We asked whether other TDP-S6 inclusion body components are also in the arsenite-induced stress granules of TDP-43 overexpressing cells. This was indeed the case for G3BP (**Figure 8A**), eIF4A1 (**Figure 8B**), and PABPC1 (**Figure 8C**). Therefore, although they differ in size, cytoplasmic inclusion bodies associated with TDP-S6 overexpression and cytoplasmic arsenite-induced stress granules which include overexpressed TDP-43 both appear similar in terms of the presence of RNA-binding proteins that co-aggregate with TDP-S6. In other words, a tight, multi-hubbed interaction network between TDP-43 and other RNA binding proteins in stress granules is activated by cellular stress, but is not required for the same interactions occurring between these proteins and the TDP-S6 isoform.

### TDP-43 and TDP-S6 Colocalize with Nuclear Splicing-associated RNA and DNA Binding Factors

Three paraspeckle proteins (RBM14, NonO and PSF) involved in pre-mRNA splicing and transcriptional repression [47], [48] were significantly enriched in the insoluble fraction from both the TDP-43 and TDP-S6 models (**Table 1**). However, all three paraspeckle proteins remained exclusively in nuclear speckles and largely colocalized with overexpressed TDP-43 (**Figure 9**), and with some portion of nuclear but not cytoplasmic TDP-S6 in representative images (**Figure S2**). We conclude that proteins coenriching in the insoluble fraction of the overexpression cell models, particularly other predominantly nuclear proteins that are not overexpressed, are likely to exist in a heterogeneous population of structures that contribute to the TDP-43 and TDP-S6 aggregate proteome, potentially partly due to indirect effects of TDP-43 overexpression. These data also highlight the heterogeneity of TDP-S6 localization, varying from mostly cytoplasmic in large aggregates (**Figures 1A** **,** **3A, 3B, 3C**) to mostly nuclear in some cells (**Figure S2**). In the case of the putative PSF-TDP-S6 interaction, the relative lack of substantial colocalization in contrast to that seen for nuclear RBM14 and TDP-S6 is noted. In combination with the finding of biochemical enrichment in the detergent insoluble fraction, this observation may suggest that an indirect effect of TDP-S6 overexpression is to increase PSF association with chromatin; PSF-chromatin interaction is thought to promote dynamic changes in transcription coregulator complexes, thereby affecting gene-specific transcription rates [**49**].

**Figure 12:**
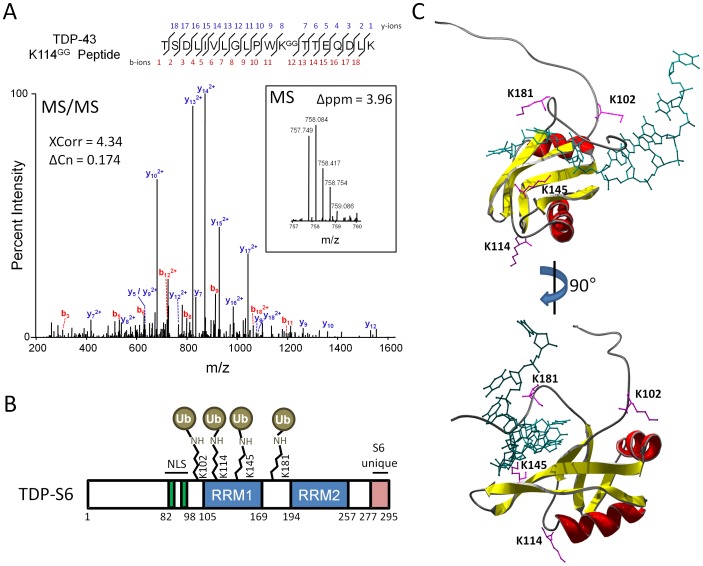
Identification of ubiquitin sites on TDP-43 by mass spectrometry. (**A**) Trypsin digestion of ubiquitin conjugates generates a di-glycine tag (GG), with a monoisotopic mass of 114.0429 Da) on ubiquitin-modified lysine residues, producing unique MS/MS spectra that can be matched by SEQUEST. Searching against a concatenated target-decoy database followed by manual validation, three sites of lysine ubiquitination (K102, K114 and K181) were mapped to TDP-43 in the TDP-S6 insoluble proteome. A fourth site (K145) identified later is also shown. (**B**) Ubiquitination site context in the linear domain structure of TDP-S6 is shown. (**C**) Two views rotated about the y-axis of a three-dimensional model of RRM-1 docked to HIV TAR ssDNA (blue) modified from Kuo, et al [17]. K102, K114, K145, and K181 side chains are labeled and explicitly shown (purple) in the context of the RRM-1 domain structure. The closest ε-amino group to the nucleic acid backbone (3.1 Å) was K181, which resides in the loop that follows the RRM1 C-terminus.

### Methylation of Constitutively Detergent Insoluble RGG Motif-containing Proteins Increases with TDP-43 or TDP-S6 Overexpression

Beyond generating and validating a list of interaction partners in an aggregate-enriched fraction of overexpressing cells to complement existing TDP-43 interactomes [23], [24], [29], we asked whether our MS/MS spectra harbored any valuable information about differential PTMs within the aggregate-enriched proteome of TDP-43 or TDP-S6 overexpressing cells. In addition to RRM domains, a second common RNA-binding motif, the glycine-rich Arg-Gly-Gly (RGG) motif, is present in 9 proteins coaggregating with TDP-43 or TDP-S6 in **Table 1**. Canonical RGG motifs in a number of heteronuclear ribonucleoproteins (hnRNPs) of classes A, B, and C enable them to act as RNA packaging factors, and these hnRNPs are thought to act on mRNA as histones do on DNA [50]. Interestingly, the reported hnRNP and TDP-43 interaction partner FUS/TLS that has been genetically and/or pathologically linked to ALS [51], [52] and FTLD [53] harbors a RGG motif. RGG motifs are frequently asymmetrically dimethylated on the arginine residue such that hydrogen-bonding with the RNA backbone of specific targets is interrupted, and perhaps this enables a nonspecific mRNA binding mode [54]. Therefore, we searched for mono and dimethylation on arginine-containing tryptic peptides and quantified them in the detergent insoluble proteomes already sequenced for mock, TDP-43, or TDP-S6 transfected HEK-293 cells. Intriguingly, RGG motifs were in the major dimethylarginine-containing peptides found (**Table 4**). The relative level of the modified peptides was normalized to the protein expression level (for unmodified peptides); this normalization excluded a net increase in what appears to be constitutive methylation of some proteins in the detergent insoluble fraction such as hnRNP A0. However a number of other dimethyl-RGG-containing proteins showed substantial increases in dimethylation with TDP-43 or TDP-S6 transfection. These proteins include transformer-2, FUS, hnRNP U, hnRNP A/B, and THO complex subunit 4. For example, the MS/MS spectra for coeluting peptides identifying hnRNP A/B dimethylated at R322 are shown in **Figure 10A** (transfected detergent insoluble fraction, *upper panel*, and detergent insoluble heavy internal standard, *lower panel*). Comparison of the MS spectra for differences in light/heavy ratios of this peptide in the mock, TDP-43, and TDP-S6 mixtures in **Figure 10B** show an unnormalized increase in the modified peptide in the TDP-43 overexpressing insoluble fraction. The MS spectrum for a representative unmodified peptide for hnRNP A/B sequenced in heavy and light forms in all three mixtures displays little change in the same mixtures (**Figure 10C**). Thus, the methylation status for a select subset of RGG motif-containing proteins which are intrinsically present in the detergent insoluble fraction may be responsive to the total levels of TDP-43 or TDP-S6 in the cell or minimally, their levels in the detergent insoluble biochemical fraction. We confirmed this result and localized grossly increased dimethyl arginine in the nucleus of cells with particularly high levels of overexpressed TDP-43 (**Figure 11**), so it is likely that the specific increases measured for the population reflect larger increases occurring in a relatively small fraction of transfected cells in the model. Lysine residues were also searched for selective changes in mono-, di-, and trimethylation in the same detergent insoluble fraction MS/MS data set, but no substantial changes were observed after normalization (**Table S3**).

**Figure 13:**
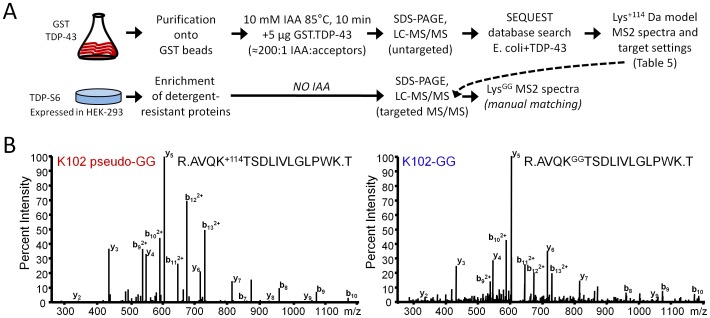
Dialkylated peptide standard development for discovery and validation of ubiquitinated lysine peptide MS/MS spectra. (**A**) Workflow for generating pseudo-ubiquitinated peptides. Peptides for all spectra generated and detected are listed in Table 5. (**B**) Comparison of MS/MS fragmentation spectra for the K102 pseudo-GG containing peptide (left panel) and the true K102-GG peptide (right panel). Spectra of all true lysine ubiquitination site peptides, side-by-side with those for pseudo-GG peptides, are given in Table S5.

**Table 5:** Dialkylated reference peptides for determination and validation of ubiquitin attachment sites on TDP-43.

Site of TDP-43attachment to ubiquitin	Peptide Sequence	Charge	XCorr	ΔCn	MatchedIons	M+HPrecursor	Targeted MS/MSMethod Segments*
**K84**	R.K^+114^M^Ox^DETDASSAVK.V	2	2.31	0.35	14/22	1411.6369	**1**
**K84**	R.K^+114^MDETDASSAVK.V	2	2.79	0.49	13/22	1395.6420	
**K95**	K.M^Ox^DETDASSAVK^+114^VK.R	2	3.95	0.14	19/24	1510.7053	
**K95**	R.KMDETDASSAVK^+114^VK.R	2	4.01	0.10	18/26	1622.8054	
**K181**	K.LPNSK^+114^QSQDEPLR.S	2	3.46	0.49	16/24	1625.8241	
**K160**	R.FTEYETQVK^+114^VM^Ox^SQR.H	2	3.53	0.54	17/26	1875.8905	**2**
**K160**	R.FTEYETQVK^+114^VMSQR.H	2	4.03	0.61	20/26	1859.8956	
**K145**	K.TGHSK^+114^GFGFVR.F	2	2.38	0.55	15/20	1306.6651	
**K136**	K.EYFSTFGEVLM^Ox^VQVK^+114^K.D	2	3.84	0.07	18/30	2035.0204	
**K102**	R.AVQK^+114^TSDLIVLGLPWK.T	2	5.34	0.56	23/30	1882.0796	**3**
**K114**	K.TSDLIVLGLPWK^+114^TTEQDLK.E	2	3.97	0.25	23/36	2271.2230	
**K224**	R.EFFSQYGDVM^Ox^DVFIPK^+114^PFR.A	2	2.39	0.55	16/36	2452.1641	
**K121**	K.TTEQDLK^+114^EYFSTFGEVLM^Ox^VQVK.K	3	4.13	0.54	27/84	2722.3280	**4**
**K121**	K.TTEQDLK^+114^EYFSTFGEVLMVQVK.K	3	3.17	0.42	21/84	2706.3330	

* Peptides are listed by retention time/elution order from reverse phase C18 nano-LC column.

The significance of changes in RGG motif methylation stoichiometry in the detergent-insoluble fraction of TDP-43 and/or TDP-S6 overexpressing cells is subject to additional questions. For example, since the identified RNP proteins with increasing RGG methylation are not in the target aggregate proteome that changes with TDP-43 or TDP-S6 overexpression, this may be a response to TDP-43 overexpression that alters affinity for (possibly promoting) RNA binding in RNPs at a more global level outside of TDP-43 aggregates. Moreover, increased occupancy of the methylated form of RGG motifs by RNA that accumulates in response to TDP-43 overexpression could drive detergent insolubility and increase the apparent stoichiometry of the methylated vs. unmethylated forms of these proteins in the detergent insoluble fraction. Alternatively, TDP-43 has been found to extensively bind to exons of arginine methyltransferase PRMT2/HRMT1L1 mRNA [29] and TDP-43 overexpression may thus selectively increase PRMT2 translation, and PRMT2 interacts with hnRNPs [55]. Regardless, we have identified the possibility that RNA binding in this handful of proteins would be altered by methylation in response to TDP-43 overexpression and/or aggregation. Future studies may thus be warranted to examine if this phenomenon is relevant to mechanisms of pathogenesis occurring during TDP-43 proteinopathy in neurodegenerative disease. It is also possible that differential methylation at these or similar sites plays a role in the remodeling of RNPs in response to stress related to RNA accumulation due to the presence of exogenous, overexpressing genes. With regards to oxidative stress, we looked for (but did not find) asymmetric dimethyl arginine in proteins accumulating within cytoplasmic TDP-S6 inclusion bodies or arsenite-induced stress granules.

**Figure 14:**
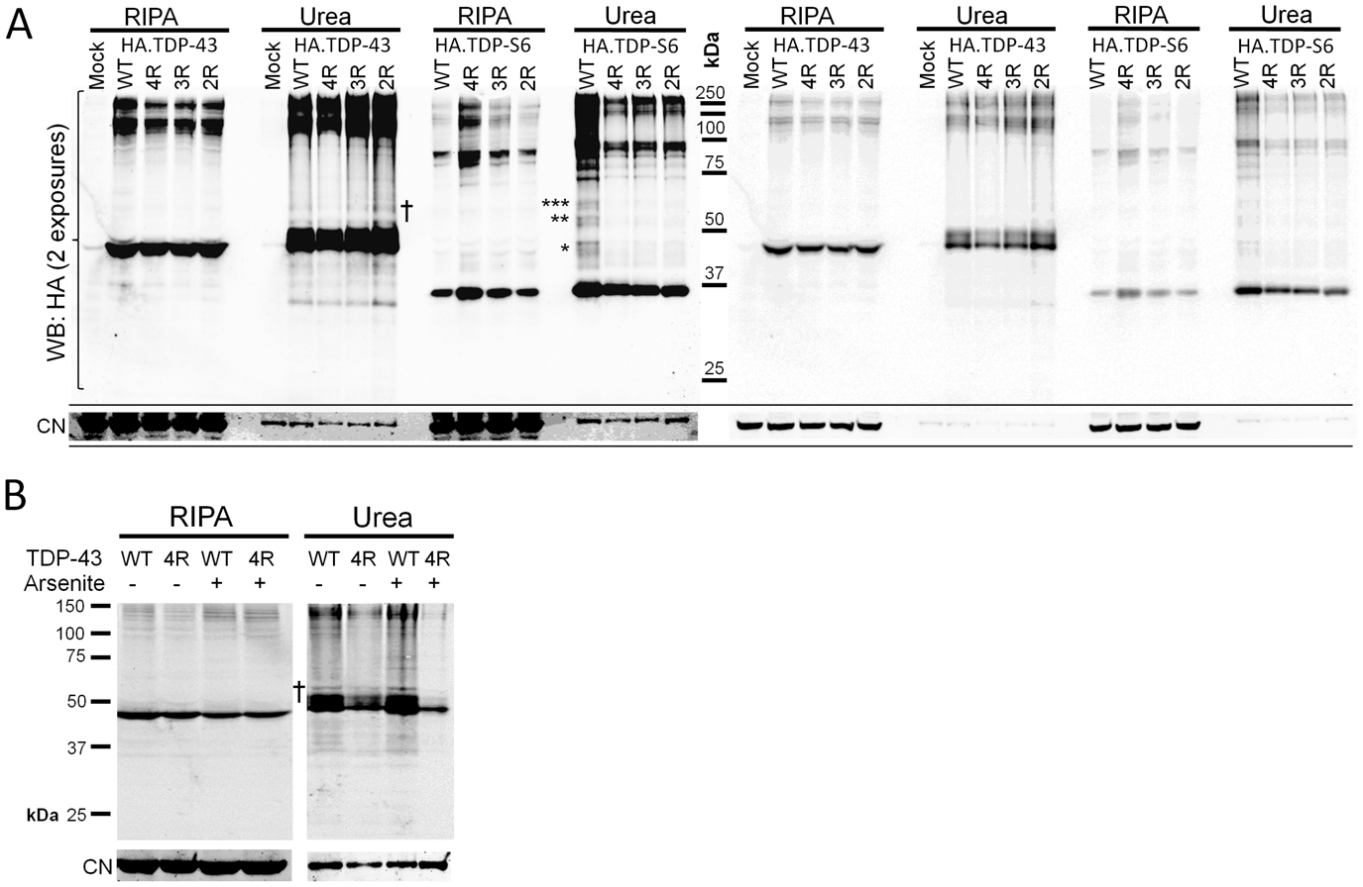
Biochemical fractionation of TDP-43 and TDP-S6 into detergent soluble and insoluble fractions is altered by the cumulative mutation of the four identified RRM1 lysine ubiquitin attachment sites on TDP-43 and TDP-S6. (**A**) Western blot of the N-terminal HA tag (upper panel) on overexpressed TDP-43 or TDP-S6 in HEK-293 extracts indicates a change in high molecular weight species and ubiquitinated species of the overexpressed proteins. Right panels give a lower exposure of the same blot. Expected positions of monoubiquitinated TDP-43 (†), and mono-, di- and tri-ubiquitinated TDP-S6 (*, **, ***) are indicated. 18 µg of RIPA extract and 3 µg of RIPA-insoluble (urea) extracts were loaded. WT, wild type; 4R (K102R/K114R/K145R/K181R); 3R (K102R/K114R/K181R); 2R (K102/K181R for TDP-43 and K114R/K181R for TDP-S6). (**B**) Detergent-fractionated HEK-293 cell extracts from cells transfected with equal amounts of WT or 4R TDP-43 were immunoblotted for the N-terminal HA tag. Where indicated, cells were treated for 1 h with 0.5 mM sodium arsenite. †, the expected position of monoubiquitinated TDP-43 is indicated. Western blot signal for calnexin (CN, 90 kDa) is provided as a loading control.

### Identifying Ubiquitination Sites on TDP-43 with TDP-S6

Ubiquitination alters the fate of proteins, expediting degradation or changing activity and trafficking, and thereby plays central roles in neurodegeneration and cancer [56], [57]. Identification of ubiquitination sites on specific lysine residues of a protein enables subsequent molecular studies that determine the effects of ubiqitination on the protein fate. However, discovery of the lysine residues to which ubiquitin is tethered remains a bottleneck in this process. Mass spectrometry is the one method capable of directly ascertaining this information in many cases where the modified lysine residue retaining an ubiquitin remnant (glycine-glycine or GG) resides in a region amenable to MS/MS sequencing following trypsin digestion and missed cleavage at the GG-modified lysine residue. The peptide harboring the modified residue must be of length and chemical character so that it can be sequenced by the MS/MS system chosen (i.e. a proteotypic peptide). Ubiquitin-like modifications, neddylation and ISG15 modification of proteins at lysine, also produce a Gly-Gly tag. However, NEDD8 and ISG15 are absent from the list of proteins co-enriched with either TDP-43 or TDP-S6 in the detergent insoluble fraction while ubiquitin is coenriched 2.5 fold with TDP-S6 (Table 1), making it possible to conclude with confidence that any Gly-Gly tagged TDP-S6 peptides identified would be most likely due to ubiquitination.

Detergent insoluble TDP-S6 on immunoblots is found in high molecular weight polymers (**Figure 1B**), and TDP-S6 colocalizes with polyubiquitin in aggregate structures by immunofluorescence [7]. Therefore, we queried our MS/MS spectra from mock, TDP-43 and TDP-S6 detergent insoluble fractions using the SEQUEST database search algorithm for TDP-S6 peptides that were modified by a Gly-Gly tryptic remnant of the ubiquitin C-terminus on lysine residues [58]. Mapping ubiquitin attachment sites on TDP-43, TDP-S6 or any ubiquitin substrate is challenging due to Ub-mediated turnover and a very low ratio of modified to unmodified substrate. Exploiting high resolution of 60,000 FWHM and mass accuracy (≤10 ppm) of the LTQ-Orbitrap, and the high enrichment factors for both TDP-S6 and ubiquitin in the TDP-S6 detergent insoluble fraction, it was possible to initially match three MS/MS spectra using SEQUEST with a concatenated target-decoy database, and validate each spectrum by manual inspection. One MS/MS spectrum for the K114-Gly-Gly peptide from TDP-43 or TDP-S6 in the TDP-S6 insoluble proteome is provided in **Figure 12A**. The spectra for two other ubiquitin attachment site peptides, K102-Gly-Gly and K181-Gly-Gly, are provided in **Table S5**. TDP-S6 sequesters endogenous TDP-43 in TDP-S6 aggregates [7], thus, these identified sites may be on TDP-S6, endogenous TDP-43, or both. The three sites and a fourth (K145) identified during further validation (see below results section) are shown in context of the TDP-S6 domain map (**Figure 12B**) and a nuclear magnetic resonance structure of TDP-43 RRM1 (**Figure 12C**). Interestingly, all four ubiquitin conjugation sites are conserved in TDP-43 homologs from species as divergent as zebrafish; K114, K145, and K181 are conserved in *D. melanogaster*, and K145 and K181 are still conserved in the closest *C. elegans* TDP-43 homolog.

### Ubiquitination Site Discovery and Validation Employing a Dialkylated Lysine Standard

To further validate the ubiquitination sites found and to search for other GG-remnant peptides missed by shotgun LC-MS/MS, we chemically derived recombinant purified glutathione S-transferase (GST)-TDP-43 with two alkyl groups mimicking the GG remnant on lysine with regards to mass and miscleavage upon tryptic digestion (producing pseudo-GG peptides and pseudo-GG MS/MS spectra [18]). These spectra and relative retention times were then used as an external reference (**Table 5**), which enabled us to develop a targeted LC-MS/MS method that we applied to an additional biological replicate of TDP-S6-transfected detergent insoluble extracts which were kept free of alkylating agent (**Figure 13A**). This approach validated the three spectra obtained and permitted serendipitous identification of an additional, fourth GG peptide on TDP-S6 or TDP-43 at K145. Each major MS/MS fragment ion from the K102 peptide that has lysine with a 114.0429 Da shift due to heat-induced dialkylation (**Figure 13B**, left panel) corresponds to a mass-identical fragment from the true ubiquitin-modified K102-GG peptide (**Figure 13B**, right panel). For matches between the other MS/MS spectra, see **Table S5**. This method is widely applicable to the discovery of ubiquitination sites on any protein of interest.

It is of particular interest that all four ubiquitin attachment sites occur within or near RRM1 of TDP-43. We note that there are four additional potential ubiquitination sites in RRM1 that do not fall within proteotypic tryptic peptides and could not be sequenced (K136, 137, 140, and 176). Moreover, a recent proteome-wide study in HEK293T cells found K102 and K114 as well as K160 to be ubiquitinated [59], and these sites were further confirmed in a second recent high-throughput study which also identified ubiquitination dependent upon various enhancing treatments on as many as 11 distinct TDP-43 lysine residues [60]. So, it is possible that RRM1 lysine residues in general are preferential, if not interchangeable, ubiquitination targets. The K160 ubiquitination product was reported to occur in response to proteasome inhibition, whereas no proteasome inhibition was used in our studies.

### Phenotypes of TDP-43 and TDP-S6 Ubiquitin Site Mutants

We hypothesized that RRM1 ubiquitin attachment could limit the degrees of freedom of motion between RRM1 and other parts of the protein, thereby affecting intramolecular interaction between RRM1 and RRM2 in a TDP-43 monomer. Intra- and intermolecular interactions between RRM domains have been reported in a number of dual-RRM containing factors, and one structural study found that TDP-43 RRM2 intermolecular interactions form a thermostable polymer [17], consistent with evidence for intermolecular cysteine disulfide formation across RRM2 domains [46]. To test if RRM1 ubiquitination alters the solubility or oligomerization propensity of TDP-43, we mutated the four potential ubiquitin-bearing lysines that we identified in TDP-43 and TDP-S6 RRM1 (K102R/K114R/K145R/K181R, “4R”) and compared the efficiency of RIPA detergent extraction of HEK-293 cells overexpressing wild type (WT), 4R, 3R, and 2R mutants of TDP-43 and TDP-S6 by western blotting extracts equally loaded by protein weight as determined by BCA assay. The result shown in **Figure 14A** [western blot for N-terminal HA tag, with calnexin (CN) signal for loading control] indicates that TDP-S6 solubility in RIPA increases considerably for the 4R mutant relative to wild type TDP-S6, and that the TDP-S6 4R mutant has decreased detergent-insoluble polymerized TDP bands at 90–100 kDa and >150 kDa, even relative to the 3R and 2R mutants. TDP-43 4R mutations modestly decreased high molecular weight polymerization in the both the RIPA-soluble and -insoluble fractions, and may have an additional negative effect on accumulation of the HA-tagged phosphorylated species at 50 kDa and higher molecular weight bands, which we confirmed in a replicate blot with anti- pS409/pS410-TDP-43 (**Figure S3**). Even though deubiquitinase activity was inactivated by IAA, we noted that in WT TDP-43, there is only a faint steady state band at the expected location of monoubiquitinated (or SUMOylated) HA.TDP-43 († in **Figure 14A**) or HA.TDP-S6 bands corresponding to mono, di-, and tri-ubiquitinated species (*, **, and *** in **Figure 14A**). Consistently, these bands are further diminished by K-to-R substitutions at the 4 ubiquitination sites identified (**Figure 14A**, left panel). We conclude that modification of TDP-43 by ubiquitin or ubiquitin-like proteins, and particularly of TDP-S6, may induce or follow polymerization into high molecular weight detergent-insoluble species.

We asked whether the decrease in polymerization of TDP-43 with ubiquitination site mutation might correspond with a resistance to oxidative polymerization of the mutant protein in response to oxidative stress (arsenite, [46]) in the detergent-insoluble fraction of HEK-293 cells. To answer this question, we repeated transfection of WT and 4R TDP-43, and 1 hour before harvest, we treated cells with 0.5 mM arsenite. Indeed, accumulation of detergent-insoluble ubiquitinated and polymerized TDP-43 was decreased not only basally, but also was strikingly absent in the 4R mutant cells detergent insoluble fraction following arsenite exposure (**Figure 14B**, lower left panel). Finally, to address whether the effects of decreased ubiquitination can be extended to cell types other than HEK-293 cells, we performed a similar experiment in HeLa cells. In addition, we asked whether the decreases in ubiquitination and polymerization of TDP-43 or TDP-S6 K-to-R mutants were resistant to an additional treatment which promotes increases in TDP-43 ubiquitination (USP14 deubiquitinase inhibition plus proteasome inhibition 6 h before harvest of cells, [61]). **Figure S4** demonstrates that indeed, the 4R RRM1 ubiquitination site mutant of TDP-43 has decreased accumulation of detergent-insoluble high molecular weight species of the overexpressed protein exposed to oxidative or proteolytic stress in HeLa cells. The residual band at the expected molecular position of monoubiquitinated TDP-43 4R mutant († in **Figure S4**) is consistent with the existence of additional lysine residues on TDP-43 suitable for ubiquitin or ubiquitin-like modifier conjugation during stress, as already reported in global proteomic studies [60].

Further studies are warranted to identify E3 ubiquitin ligase(s) that target RRM1 of TDP-43 during pathogenesis. If inhibition of these enzymes can be achieved, disease course may be altered. Evidence further suggests that ubiquitin dynamics on RRM1 of TDP-43 may also coordinate its participation in aggregates with oxidized polymers. This data suggests an intriguing role for ubiquitination of TDP-43 RRM1 in its participation in stress granules and paraspeckles, with potential effects on nucleation, assembly or disassembly, and thereby would respectively affect translation and transcription-coupled splicing in response to cellular conditions inducing such ubiquitination. Precedent for a role of ubiquitination of an RNA-binding protein affecting translation in neurons at dendrites with effects on learning and memory was recently published by the Kandel group [62], and as TDP-43 RNA transport and translational control has also been shown to respond to neuronal activity [30], it will be key to identify if ubiquitination of TDP-43 plays a similar regulatory role.

### Concluding Remarks

This study presents significant advances in the characterization of TDP-43-associated PTMs and potential effects of TDP-43 PTMs on phenotypic changes in proteins with RRM and RGG RNA-binding domains. The four sites of direct ubiquitin conjugation to TDP-S6 at residues K102, K114, K145, and K181 are conserved with the RRM1 fold in TDP-43 homologs, consistent with a yet unappreciated role or roles in TDP-43 function. The TDP-S6 target aggregate proteome, which accumulates non-degradation-associated K63 and to a lesser extent K48 ubiquitin linkages [7] contrasts with the enrichment of SUMO2 and/or 3 and its colocalization with overexpression of nuclear TDP-43 [7]. We speculate that the small fraction of full length TDP-43 transiting outside the nucleus [3] would be exposed to functional modulation by a cytoplasmic E3 ubiquitin ligase. In the case of ubiquitination on RRM1, data from this study suggests that this PTM has a role in promoting TDP-43 self-interaction in detergent-insoluble aggregates, particularly in response to oxidative stress (**Figure 14**), and these aggregates include other RNA-binding proteins, the solubility of which might also be affected by TDP-43 ubiquitination. The context of attachment sites in the structure of RRM1 (**Figure 12C**) suggests that RNA binding by TDP-43 itself would likely be modified by ubiquitination.

Ubiquitin conjugate accumulation, insolubility of TDP-43 in aggregates or inclusions, and cleavage fragments of TDP-43 have been found to occur during the pathogenesis of TDP-43 proteinopathies including ALS and FTLD, as well as in diseases not previously associated, including Alzheimer disease, Machado-Joseph disease, and Parkinson disease [63]–[73]. In this study, we have identified an intersection between the interactions of RNA binding proteins and ubiquitination, and our data are consistent with a model wherein this intersection lies just upstream of translation and/or translational inhibition. The choice for TDP-43 to deliver and release mRNA to translation preinitiation complexes or to inhibit mRNA translation could absolutely depend on the presence or absence of conditions to which TDP-43 conformation is sensitive, such as oxidative redox balance [46] or the accumulation of similar aggregate-prone proteins in a destabilized, or even prion-like state. Ubiquitination of TDP-43 RRM1 appears to promote or coincide with such a state, and it is likely not by chance that many of the co-aggregating RNA-binding proteins we detected also contain prion-like domains [74]. A timeline of kinetics for TDP-43 ubiquitination under different conditions, splice variant expression, and participation in distinct cytoplasmic and nuclear aggregates in future studies will provide further insight into mechanisms of pathogenesis in neurodegenerative diseases.

## Supporting Information

Figure S1 **Protein groups in the background detergent insoluble proteome and those significantly changed in the TDP-43 and TDP-S6 target aggregate proteomes.** Protein categories defined by DAVID version 6.7 were graphed as a percentage of the population of the 585 proteins identified in Table S1 (grey bars), and for the 41 significantly changing proteins in Table 1 (red bars, increasing; green bar, decreasing).
(TIF)

Figure S2 **Costaining of Nuclear RNA-binding proteins with TDP-S6.** **(A, B)** HA.TDP-S6 (red) colocalization with nuclear PSF **(A)**, arrow, or RBM14 **(B)** in distinct subnuclear structures in representative cells. PSF and RBM14 staining are shown in green. Scale bars, 10 µm.
(TIF)

Figure S3 **Phospho Ser409/Ser410 TDP-43 decreases in the insoluble fraction with ubiquitination site removal via mutation of lysine to arginine.** Western blot of the same membrane for HA.TDP-43 (anti-HA, left panel) and phospho Ser409/Ser410 of TDP-43 (right panel).
(TIF)

Figure S4 **Quadruple TDP-43 ubiquitin site mutant** **is resistant to oligomerization following oxidative or proteolytic stress in HeLa cells.** Detergent-fractionated HeLa cell extracts from cells transfected with equal amounts of WT or 4R TDP-43 and treated as indicated for 1 h with sodium arsenite or 6 h with MG-132 plus IU1 were immunoblotted for the HA tag. †, the expected position of monoubiquitinated TDP-43 is indicated. Western blot signal for calnexin (CN, 90 kDa) is provided as a loading control.
(TIF)

Table S1 **Quantification of 585 proteins in both experimental replicates.** All proteins quantified by light/heavy peptide ratio in both experimental replicates (insoluble/insoluble and insoluble/soluble experimental/standard light/heavy mixtures) are listed. Standard deviation across all quantified peptides of each protein is given along with the number of quantified peptides, and average signal to noise ratio of the quantified peptides for each of six light/heavy mixtures. Mean normalization factors for recentering population data are given on the darkened header row above each of the six log2(light/heavy) data columns.
(XLS)

Table S2 **All proteins and peptides identified by LC-MS/MS of mock, TDP-43, or TDP-S6 transfected detergent insoluble fractions.** Total number of spectral counts (SC), the number of unique peptides assigned on each protein, sequencing coverage (%), the mass error measured in Orbitrap (deltaMass), the SEQUEST matching scores (XCorr and deltaCn), the link to assigned spectra, and where calculated, extracted ion current intensity expressed as signal-to-noise ratio for paired light and heavy peptides in mixtures are listed. The labeled heavier K or R residues are marked (@, #).
(ZIP)

Table S3 **Lysine methylation site peptides identified and quantified in the TDP-43 and TDP-S6 insoluble proteome of HEK-293 cells.** Histone and elongation factor Tu methylation sites were identified without any quantified change due to TDP-43 or TDP-S6 overexpression. Note that H3 methylation sites are numbered on the mature histone without Met1. Ion suppression affected the four WT TDP-43 histone H3 XIC intensities in a single LC-MS/MS run relative to the comparable TDP-S6 and Mock runs. This suggests that extra caution must be taken when quantifying peptides without an internal standard.
(XLS)

Table S4 **Identification of methylated and ubiquitinated proteins and peptides.** All peptides identified by MS/MS to harbor PTMs in this study are listed with the mass error measured in Orbitrap (deltaMass), the SEQUEST matching scores (XCorr and deltaCn) and matched ion count. The labeled K or R residues are marked as follows: dimethyl-Lys (@), methyl-Lys ∧(), dimethyl-Arg (#), Lys-epsilon-amino-GG (ubiquitination, &).
(XLS)

Table S5 **Comparison of pseudo-GG peptide MS/MS spectra obtained by LC-MS/MS of IAA-alkylated GST-TDP-43 and** ***bona fide*** **TDP-43 or TDP-S6 GG peptide spectra.** Sequest matched ions are shown on MS/MS spectra for pseudo-GG peptides obtained for purified GST-TDP-43. Where available to the right, representative lysine-GG peptide spectra sequenced from the urea-solubilized detergent insoluble fraction of TDP-S6 overexpressing HEK-293 cells (K102-GG, K114-GG, K145-GG, and K181-GG) are also shown to the right. The K145-GG peptide mascot spectrum and matched ions table are also inset to the right.
(XLS)
