# Supplementary figures and images for: Coaggregation of RNA-Binding Proteins in a Model of TDP-43 Proteinopathy with Selective RGG Motif Methylation and a Role for RRM1 Ubiquitination
Source: PLoS One. 2012 Jun 21;7(6):e38658. doi: 10.1371/journal.pone.0038658 (PMC3380899; doi:10.1371/journal.pone.0038658)

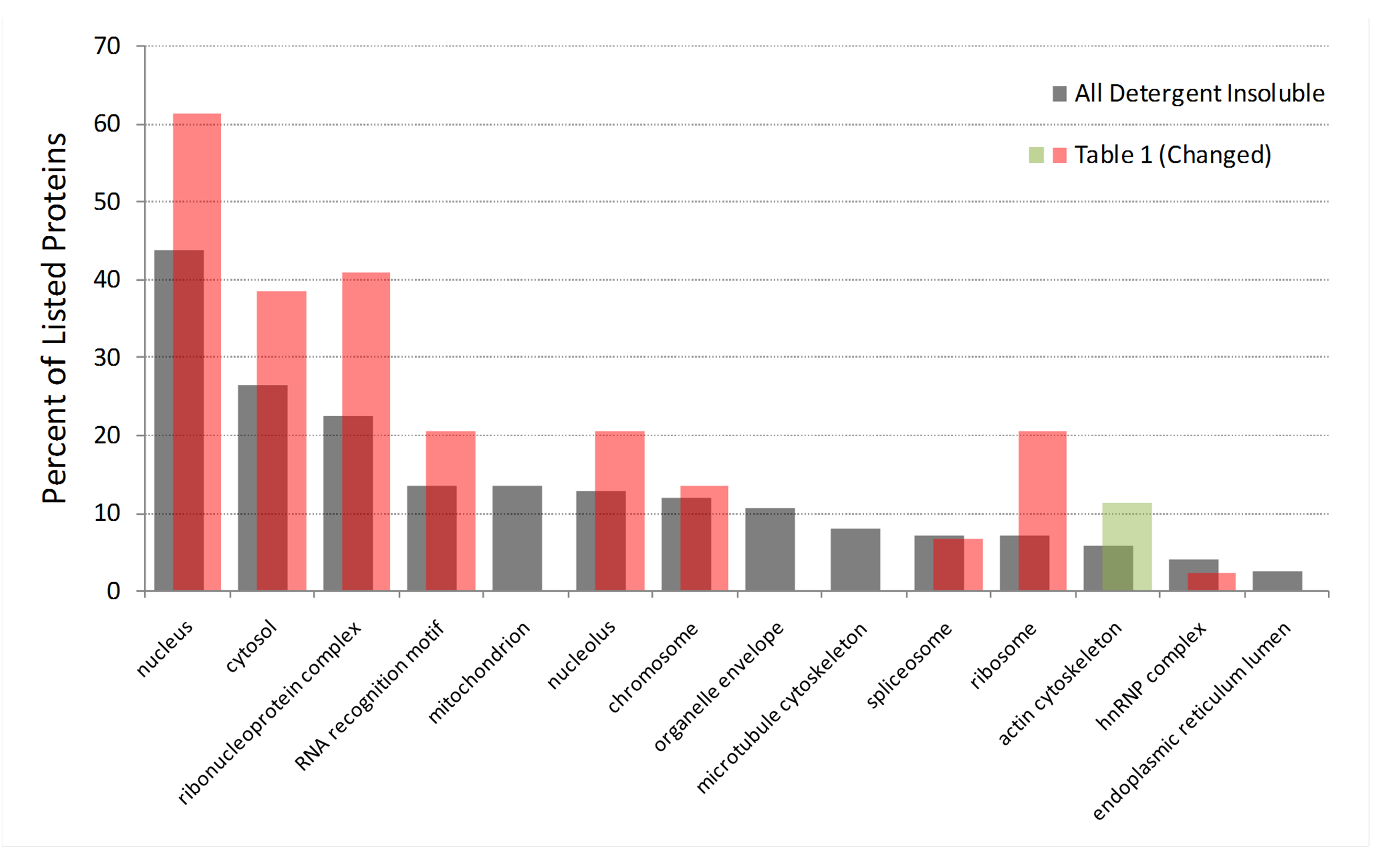

Supplement: Figure S1 — Protein groups in the background detergent insoluble proteome and those significantly changed in the TDP-43 and TDP-S6 target aggregate proteomes. Protein categories defined by DAVID version 6.7 were graphed as a percentage of the population of the 585 proteins identified in Table S1 (grey bars), and for the 41 significantly changing proteins in Table 1 (red bars, increasing; green bar, decreasing). (TIF) [file pone.0038658.s001.tif]

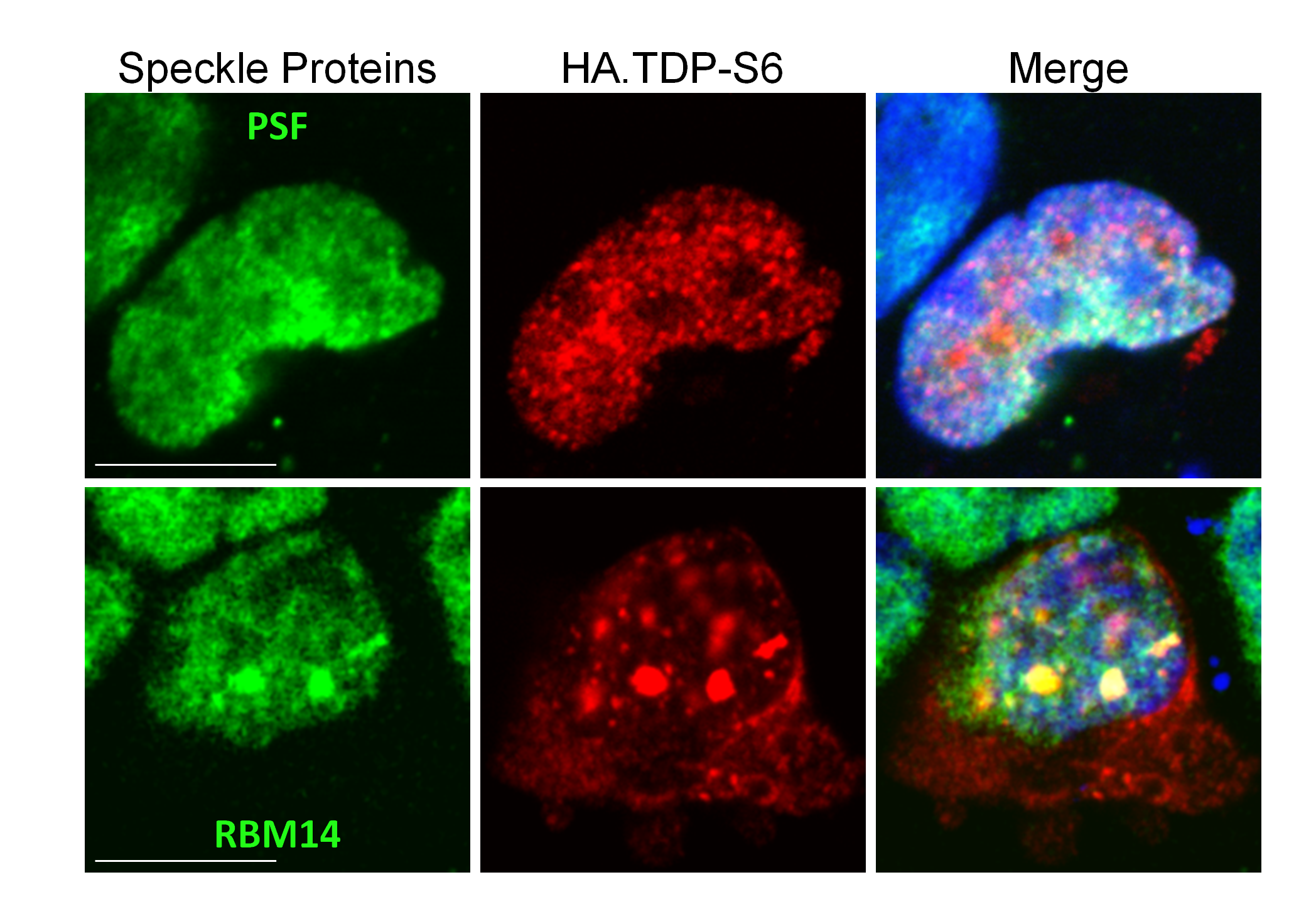

Supplement: Figure S2 — Costaining of Nuclear RNA-binding proteins with TDP-S6. (A, B) HA.TDP-S6 (red) colocalization with nuclear PSF (A), arrow, or RBM14 (B) in distinct subnuclear structures in representative cells. PSF and RBM14 staining are shown in green. Scale bars, 10 µm. (TIF) [file pone.0038658.s002.tif]

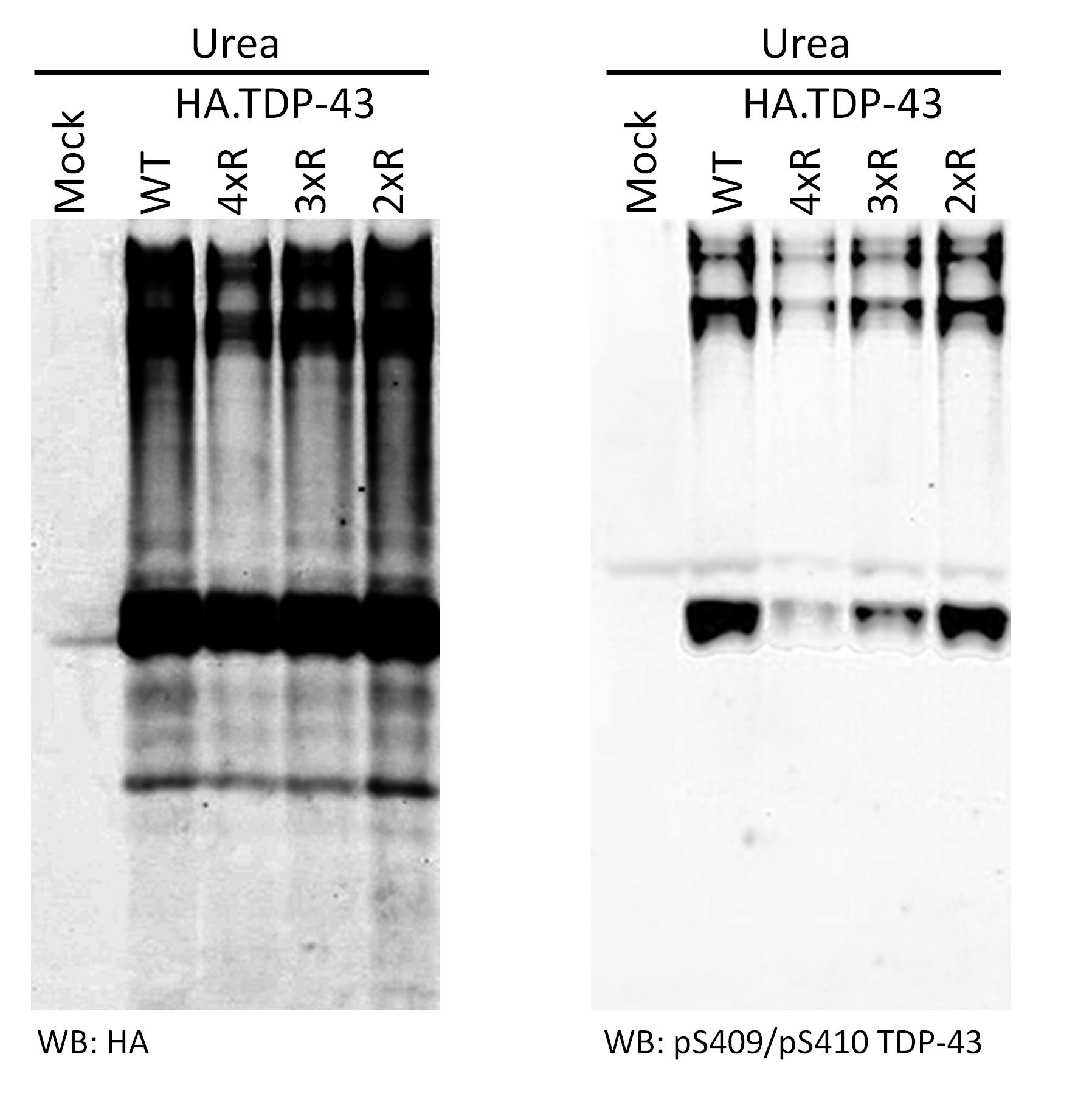

Supplement: Figure S3 — Phospho Ser409/Ser410 TDP-43 decreases in the insoluble fraction with ubiquitination site removal via mutation of lysine to arginine. Western blot of the same membrane for HA.TDP-43 (anti-HA, left panel) and phospho Ser409/Ser410 of TDP-43 (right panel). (TIF) [file pone.0038658.s003.tif]

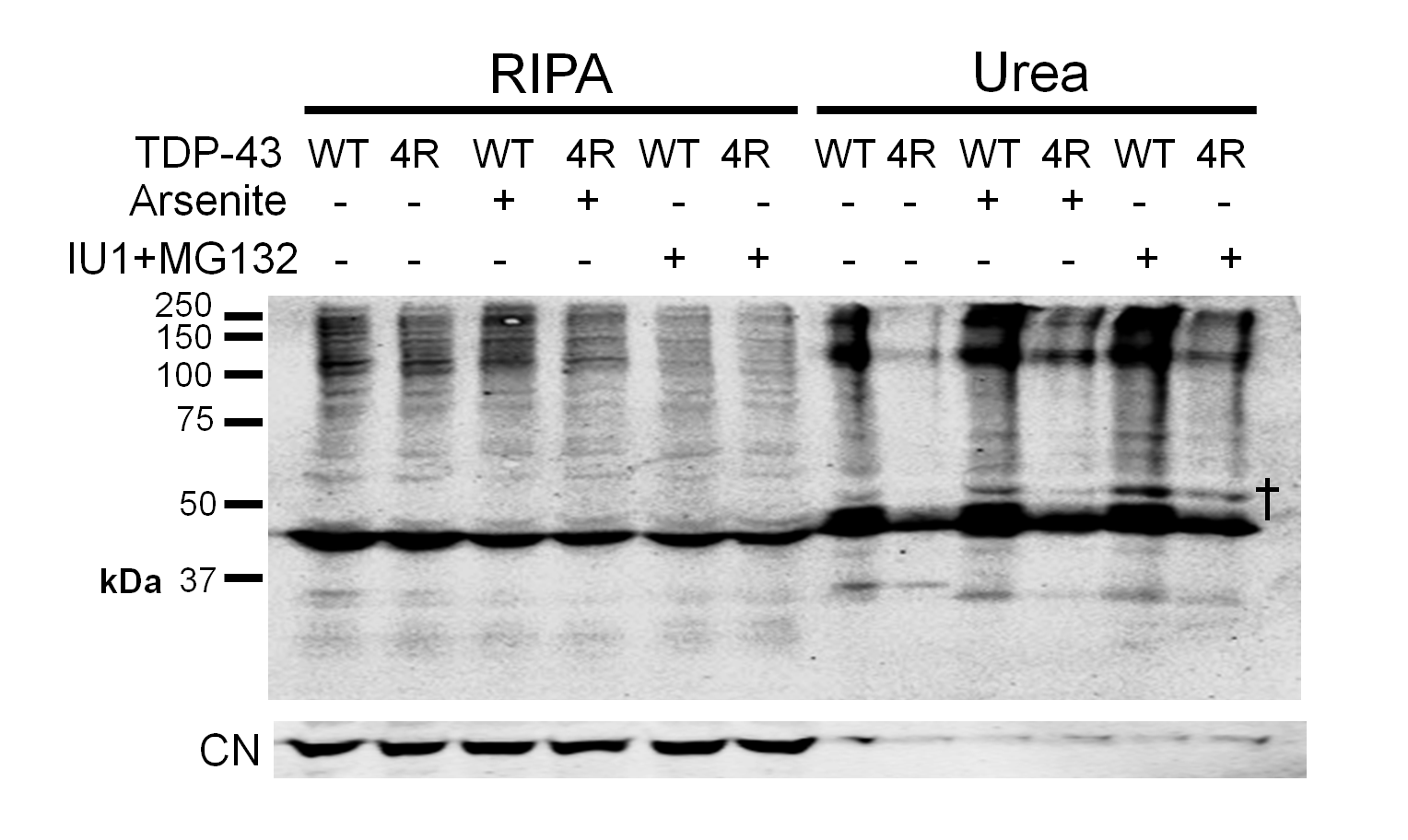

Supplement: Figure S4 — Quadruple TDP-43 ubiquitin site mutant is resistant to oligomerization following oxidative or proteolytic stress in HeLa cells. Detergent-fractionated HeLa cell extracts from cells transfected with equal amounts of WT or 4R TDP-43 and treated as indicated for 1 h with sodium arsenite or 6 h with MG-132 plus IU1 were immunoblotted for the HA tag. †, the expected position of monoubiquitinated TDP-43 is indicated. Western blot signal for calnexin (CN, 90 kDa) is provided as a loading control. (TIF) [file pone.0038658.s004.tif]
